# Proteolytic and non-proteolytic regulation of collective cell invasion: tuning by ECM density and organization

**DOI:** 10.1038/srep19905

**Published:** 2016-02-02

**Authors:** Sandeep Kumar, Aastha Kapoor, Sejal Desai, Mandar M. Inamdar, Shamik Sen

**Affiliations:** 1 Department of Biosciences and Bioengineering, IIT Bombay, Mumbai, India; 2 Civil Engineering Department, IIT Bombay, Mumbai, India

## Abstract

Cancer cells manoeuvre through extracellular matrices (ECMs) using different invasion modes, including single cell and collective cell invasion. These modes rely on MMP-driven ECM proteolysis to make space for cells to move. How cancer-associated alterations in ECM influence the mode of invasion remains unclear. Further, the sensitivity of the two invasion modes to MMP dynamics remains unexplored. In this paper, we address these open questions using a multiscale hybrid computational model combining ECM density-dependent MMP secretion, MMP diffusion, ECM degradation by MMP and active cell motility. Our results demonstrate that in randomly aligned matrices, collective cell invasion is more efficient than single cell invasion. Although increase in MMP secretion rate enhances invasiveness independent of cell–cell adhesion, sustenance of collective invasion in dense matrices requires high MMP secretion rates. However, matrix alignment can sustain both single cell and collective cell invasion even without ECM proteolysis. Similar to our *in-silico* observations, increase in ECM density and MMP inhibition reduced migration of MCF-7 cells embedded in sandwich gels. Together, our results indicate that apart from cell intrinsic factors (i.e., high cell–cell adhesion and MMP secretion rates), ECM density and organization represent two important extrinsic parameters that govern collective cell invasion and invasion plasticity.

*In vivo*, cells are known to utilize a gamut of different migration strategies for their movement. Single cell migration is the best-studied mechanism and is important in various processes including development, immune surveillance and cancer metastasis^1^,^2^,^3^. In epithelial cancers, invasion is often triggered by conversion from a cell–cell adhesion-rich ‘epithelial state’ to a cell–cell adhesion-weak ‘mesenchymal state’ in a process termed epithelial to mesenchymal transition (EMT)^4^. Collective cell migration^5^,^6^ and collective cell invasion^7^,^8^ represent a second mode of cell movement wherein cell–cell adhesions remain intact and cells move collectively^9^,^10^. During collective cell motility, a front-rear asymmetry is maintained whereby leader cells at the front row sense extracellular guidance cues through a combination of chemotaxis and haptotaxis and dictate the overall direction of movement for the cell cohort. A common feature of both single and collective cell invasion is the requirement for extracellular matrix (ECM) remodeling, achieved primarily through matrix degrading enzymes like matrix metalloproteinases (MMPs), which generate paths via ECM degradation^11^,^12^. Interestingly, inhibition of degrading machinery or blockage of cell–matrix adhesions leads to amoeboidal mode of migration wherein cells squeeze through pre-existing pores in the matrix using actomyosin contractility^13^. Thus, instead of arresting cell invasion, alterations in cell–cell adhesions, cell–matrix adhesions and proteolytic machinery lead to alterations in the strategies used by cancer cells to invade.

In addition to the molecular properties of tumour cells themselves, the ECM is itself a major determinant of the mode of cell invasion. In interstitial tissues, ECM organization can differ significantly from one location to another depending on the density and orientation of collagen I, the major constituent of the interstitial ECM^14^. During invasion, cancer cells manoeuvre through both sparse matrices comprised of randomly oriented fibres with large pore sizes, and dense matrices comprised of aligned collagen bundles with pore sizes of few microns. The tumour microenvironment itself is known to undergo drastic alterations in composition and organization during cancer progression^15^. Similar to fibrosis, many malignancies are associated with increased deposition of fibrillar collagens^16^,^17^, with increased expression of collagen I associated with increased risk of cancer metastasis^18^,^19^. In many epithelial tissues, the organization of collagen fibrils also changes from curly to linear effected by cancer cell-secreted crosslinking enzymes like lysyl oxidase (LOX)^20^. Increase in ECM density and crosslinking progressively stiffen the ECM, which in turn influences cell motility through increased integrin signalling^21^. Interestingly, integrin signalling also influences ECM remodeling via activation of MMPs, which generate migration tracks by proteolytic cleavage of the ECM through membrane-localized as well as soluble MMPs^11^,^22^,^23^. Given the wide heterogeneity in ECM organization, invading tumour cells are confronted with different extracellular structures, which in turn are likely to influence the mode of motility in multiple ways. Some of the critical ECM features influencing the mode of cell motility include confinement (set by pore size)^24^,^25^, fibre alignment (along which cells may align and migrate)^26^,^27^ and bulk properties of the matrix^20^,^21^,^28^,^29^.

As detailed in the above paragraphs, ECM density and orientation, cell polarity, cell–cell contact strength, and cell–ECM interactions interdependently control efficiency and the mode of cancer cell invasion^13^,^30^. However, due to the enormous biological and biophysical complexity of this cell–ECM ensemble, it is extremely difficult to experimentally gauge how these interactions contribute to cancer cell invasion. Hence, it seems appropriate to use mathematical and simulation tools, which have contributed significantly to our understanding of cancer^31^,^32^,^33^,^34^, to understand plasticity in cancer cell invasion. Several classes of computational models have been developed to address various aspects of cell invasion during cancer progression. Of the various types of models, discrete models, such as cellular automata, cellular Potts model (CPM) and agent based models, are particularly well suited for studying multiscale population dynamics^35^,^36^. Of special interest is the cell-based, CPM, a multi-particle lattice description, that is capable of modeling cell–cell and cell–ECM interactions^37^. In CPM, physical properties of biological entities (e.g., cell, ECM fibre, etc.) can be taken into account by modeling them as multi-pixel deformable objects and evolving their position/state as per experimentally motivated predefined rules^38^,^39^,^40^. Further, it is possible to combine these models with other mechanistic rules as well as partial differential equation (PDE)-based reaction–diffusion systems for capturing phenomena occurring at different length-scales and time-scales^37^,^41^,^42^,^43^.

Despite the existence of many models that have been developed for the purpose of understanding cancer invasion, several questions remain unanswered. For example, Bauer *et al.*^41^,^42^ used a hybrid CPM to understand the role of cell migration, growth and adhesion to understand tumour induced angiogenesis. However, the migration in their model was a result of directed chemotaxis, whereas cells can undergo migration due to self-polarization even in the absence of chemokine gradients^44^. The CPM developed by Kabla^45^ incorporates polarity and migration potential of single cells in order to understand migration patterns of cell collectives. However, the effects of cellular microenvironment, which are crucial for cancer cell invasion, were missing in this description. Most of these drawbacks were addressed by Szabo *et al.*^46^ in their concise description that takes into account all the crucial ingredients – cell–cell and cell–ECM interactions, cell polarization and ECM topography – and their role in cancer cell invasion. Modeling along similar lines was also done in refs 47, 48, 49. Despite these modeling efforts, various questions remain unanswered, and warrant explanation. For example, what is the role of cell–cell adhesion with respect to ECM remodeling and maintaining invasiveness of cancer cells? What is a more efficient mode of invasion (single or collective), and when would the cells prefer to switch from one mode to the other? How does ECM confinement and proteolytic abilities collectively dictate the mode and efficiency of cell invasion? In short, although the existing models indeed address the effect of various aforementioned factors on avascular cancer cell invasion, they do not elucidate how these factors conspire together to dictate the mode and efficiency of invasion, and what invasion strategies cells may flexibly follow upon tuning these factors^13^,^30^.

In this paper, we probe the contributions of cell–cell adhesion, MMP dynamics, ECM density and organization on the mode of cancer cell invasion, and attempt to address the issue of cell invasion plasticity^13^,^30^. Our computational framework uses CPM-based formalism to model a cell aggregate positioned at the centre of an ECM lattice of given density and organization. Cells possess intrinsic motility and history-dependent polarity (preferred direction of motion). Rule-based mechanistic formulation was used to implement MMP-mediated ECM fibre degradation. Pre-existing gaps and spaces created by ECM degradation allow for movement of cells within the lattice. Using this formulation we found that the overall speed and pattern of cell invasion depended greatly on cell–cell adhesiveness and MMP secretion rates. While greater cell–cell adhesiveness was required for collective cell invasion, higher cell–cell adhesion was also associated with less amount of ECM degradation. The increase in MMP secretion rate at intermediate fibre densities was observed to make individual invasion more effective as compared to collective cell invasion. On the other hand, high MMP secretion rates foster collective cell invasion at high fibril densities. Aligned matrices, which provide contact guidance cues to cells, serve as an exception to the requirement of high MMPs where invasion is possible even at low MMP secretion rates. Finally, we have compared some of our *in-silico* predictions with experiments by tracking the invasion of MCF-7 human breast cancer cells using sandwich cultures. Taken together, our results suggest that the interplay between cell–cell adhesion, MMP secretion rate and ECM organization, which can be thought of as intrinsic tuning parameters of cancer cells, can lead to plasticity in cancer cell invasion.

## Materials and Methods

**Figure 1:**
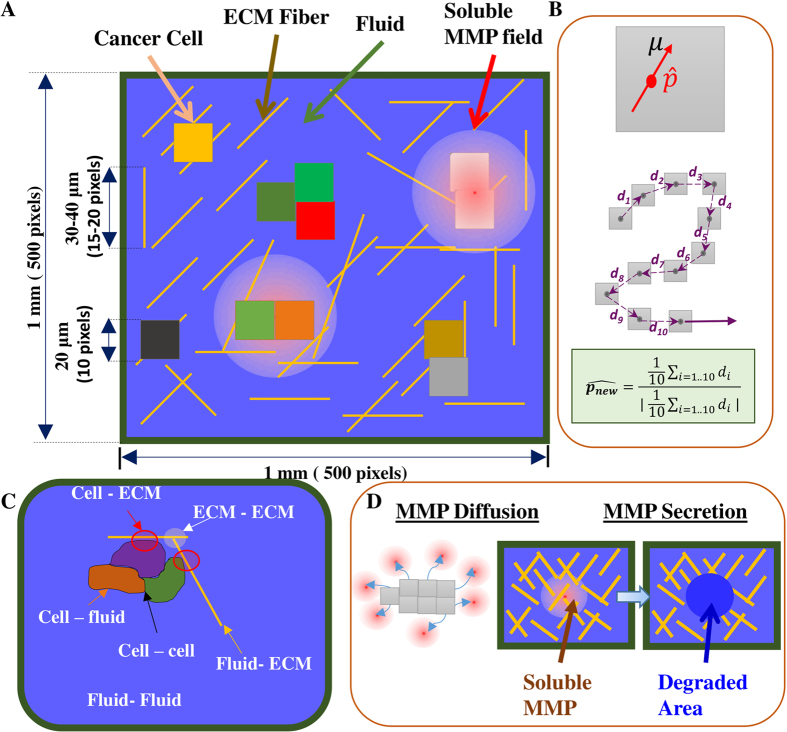
Model schematic (A) ECM was modeled as a 2D space (Ω) of 1 × 1 mm^2^. Ω is discretized into pixels of dimensions 2 *μ*m × 2 *μ*m. ECM fibres were represented by straight lines 1 pixel (2 *μ*m) thick and 30–40 *μ*m long. Each cell was represented by a 2D area ≈400 *μ*m^2^ (coloured 2D boxes). Pixels that neither belong to a cell nor to an ECM fibre were considered as fluid pixels. MMP molecules were modeled by a continuous field. (**B**) Cells were modelled as active particles with intrinsic motility. The direction of cell motility, also referred as cell polarity (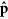), was calculated based on the average of the cell displacements in the last 10 Monte Carlo steps (MCS). (**C**) Distinct adhesion energies were defined for interfaces between adjacent entities, that is *J*_cf_ (cell–fluid), *J*_cc_ (cell–cell), *J*_ce_ (cell–ECM), *J*_ee_ (ECM-ECM), *J*_ef_ (ECM–fluid) and *J*_ff_ (fluid–fluid). (**D**) Soluble MMP molecules, secreted by cells, can undergo diffusion, and also degrade the ECM fibre, when the number of MMP molecules at any pixel increases beyond a threshold value of 1. Subsequently, the fibre is deleted from that pixel by changing the pixel type to ‘fluid pixel’.

Cellular invasion through dense ECM networks is influenced by several factors including steric hindrance from the environment, formation of migration tracks by ECM proteolysis (mediated by MMPs), self motility of cells, and adhesion energies between different entities (e.g., cell–cell adhesion, cell–matrix adhesion, etc). One of the major bottlenecks in understanding cell invasion is attributed to the multiscale nature of processes involved. While cell invasion is a cell-scale phenomenon, changes in interface energies associated with local cell movement, MMP secretion, MMP diffusion and ECM degradation occur at the sub-cellular level. Thus, for simulating cell invasion, it is important to develop a framework which combines multiple processes occurring at different length-scales and time-scales. Cellular Potts models (CPMs), also called Graner-Glazier-Hogeweg (GGH) models, are cell-based models that provide a convenient way to integrate cellular mechanics with sub-cellular reaction diffusion dynamics^38^,^39^,^40^. To tackle the multiscale phenomena of cell invasion, we have developed a Monte Carlo simulation-based CPM integrated with reaction–diffusion dynamics of MMP molecules. In our model, diffusing MMP molecules degrade ECM fibres and change cell–ECM interactions thereby integrating reaction–diffusion dynamics of MMP with GGH algorithm. Models like these, which integrate processes occurring at different length and times scales and obey different dynamics (e.g. GGH algorithm and reaction–diffusion dynamics), are referred to as multiscale hybrid models^43^,^50^,^51^. In our model, cells are placed on top of a non-degradable substrate and surrounded by an interstitial ECM network comprised of ECM fibres and interstitial fluid (Fig. 1A). The software package CompuCell3D (CC3D)^40^ was combined with custom written C++/python routines for implementing our model.

### Model components

Making use of the spherical symmetry of solid tumours/tumour spheroids, 2-dimensional (2D) computational models have been used for studying various aspects of 3-dimensional (3D) tumour invasion including 3D branching, angiogenesis and cell migration^41^,^42^,^52^. Using a similar approach in this study, we have developed a 2D model of cancer invasion, wherein ECM fibres and cells were placed inside a 2D lattice (1 mm × 1 mm size) (Fig. 1A). Making use of the symmetry property of 3D spheroids helped us in simplifying the complexity of cancer cell invasion in 3D and developing a computationally tractable model of cellular invasion, wherein we simulate invasion of a group of cells placed at the centre of our simulation grid. This setup only mimics metastatic dissemination of a group of cancer cells, and does not model cancer progression. The lattice size was selected such that each cell had to migrate at least 400 *μ*m (double the population length scale) before touching the lattice boundary. ECM was represented by a meshed network of fibres, with each fibre represented by a straight line 2 *μ*m in thickness and 30–40 *μ*m in length. In our model, all ECM fibres were aligned in a 2D plane instead of having 3D positioning. This approximation helped us to incorporate the effect of mechanical hindrance in our model with an increasing number of ECM fibres providing greater hindrance (as a cell cannot cross over a fibre without degrading it). While the fibre orientation was assumed to be random for most of the simulations (unless indicated otherwise), the effect of fibre density was incorporated in our model by changing the total number of fibres in the lattice. Cells were represented as 2D deformable surfaces of mean area ≈400 *μ*m^2^, comparable to the size of biological cells^53^. Cells are capable of changing their position and morphology over time, subject to area and perimeter constraints. While the area constraint prevents the cell area from becoming too large or too small, the perimeter constraint ensures that cells do not undergo excessive deformations. Cells were assumed to possess intrinsic motility, modeled by associating a preferred direction of migration (i.e., polarity, 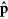) and motility strength (*μ*_0_). The polarity (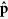) was calculated based on the last 10 displacements (Fig. 1B) as done elsewhere^45^. After placing ECM fibres and cells in the lattice, the remaining free pixels were considered as ‘fluid’ pixels, which represent the interstitial fluid between the ECM pores^42^. Adhesion energies were used to model the extent of adhesion between adjacent entities, i.e., between two ECM fibres (*J*_ee_), cell–ECM (*J*_ce_), ECM–fluid (*J*_ef_), cell–cell (*J*_cc_), cell–fluid (*J*_cf_) and fluid–fluid (*J*_ff_) (Fig. 1C). While high values of adhesion energy represents weak adhesion, strong adhesion was modeled by low values of adhesion energy.

### Interactions and evolution in CPM

Spatiotemporal evolution of the simulation lattice was governed by the random movement of individual pixels subject to transition probabilities based on the Monte Carlo method^40^. Algorithmically, during each move, two neighboring pixels were chosen randomly, with one designated as the *source* pixel and the other one as the *target* pixel. An attempt to update the lattice was made only when both the *source* and the *target* pixels represented either a cell pixel or a fluid pixel. In other words, ECM fibre pixels did not participate in the random Monte Carlo updates. Further, if both pixels belonged to the same cell (i.e., *σ*(*source*) = *σ*(*target*)), then also no changes were made to the lattice. Otherwise, the *source* pixel attempted to occupy the *target* pixel based on Monte Carlo acceptance probability. To do this, the total system energy associated with the configuration before the move (*E*_i_) and the configuration after the move (*E*_f_) were calculated as per the following equation:


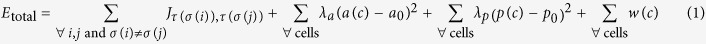


where,

*σ*(*i*): ID of the cell to which pixel *i* belongs

*τ*(*σ*): type of the cell *σ*

*J*_*τ*1,*τ*2_: boundary energy per unit length between cells of type *τ*1 and *τ*2

*λ*_*p*_: perimeter constraint of a cell

*λ*_*a*_: inverse compressibility or area constraint of a cell

*a*(*c*): current area of the cell *c*

*p*(*c*): current perimeter of the cell *c*

*a*_0_: target (preferential) area of a cell

*p*_0_: target (preferential) perimeter of a cell

*w*(*c*): energy due to active motility of the cell *c*

Between two different configurations, if Δ*E* = (*E*_f_ − *E*_i_) < 0, the proposed move was always accepted. Else, the move was accepted with a probability *p* calculated using the expression 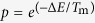. Using this dynamics, the system tries to move towards a lower energy configuration with *T*_m_ representing the likelihood of accepting a thermodynamically unfavorable move (i.e., it increases the system energy instead of decreasing). This expression of probability of accepting a move is based on the widely used Boltzmann acceptance function^54^, with *T*_m_ referred to as Boltzmann temperature. In CPM, *T*_m_ does not reflect any conventional thermal temperature; instead, it represents the strength of noise in the dynamics^45^. Low values of *T*_m_ makes the system evolution highly deterministic raising the possibility of the system getting trapped at a local minima. In contrast, a very high value of *T*_m_ causes the system to accept any proposed move. Further, owing to the fact that agitation of cell membrane in biological systems plays a role similar to that performed by temperature in thermodynamics, *T*_m_ is also called the magnitude of effective membrane fluctuations^40^,^41^,^42^. High values of *T*_m_ may lead to increase in cell body re-arrangements and motility^40^. In contrast, low values of *T*_m_ correspond to cells with very low fluctuations in cell boundary. A successful move increased the volume of the cell containing the *source* pixel and decreased the volume of the cell containing the *target* pixel by one pixel. Each Monte Carlo step (MCS) corresponded to repeating this exercise *N* times (*N* being the total number of lattice pixels that can be evolved) irrespective of whether the moves were accepted or not.

In the above expression, four different energy terms contribute to the total energy of the system (*E*_total_). These are due to adhesive interactions between different entities (e.g., cell–cell, cell–matrix, cell–fluid, etc.) and energy associated with changes in cell size, changes in cell perimeter and with the intrinsic motility of cells. Adhesive/repulsive dynamics between different entities as well as between two species of the same entity was accounted for by considering an interface energy (e.g., *J*_cc_, *J*_ce_, etc.) (Fig. 1C). Lower values of interface energies represent greater adhesion and higher values model the lower adhesion. If the cell–cell adhesion energy (*J*_cc_) is lower than the cell–matrix (*J*_ce_) or cell–fluid (*J*_cf_) energies, any move (movement of a pixel of a cell) resulting in an increase of contact surface area between two neighboring cells will be accepted as it will decrease the system energy. Conversely, if *J*_cc_ is higher than *J*_ce_ or *J*_cf_, any proposed move resulting in increase of cell–cell adhesion will be rejected due to the high energy of the proposed state^40^. Physically, lower values of *J*_cc_ represent stronger cell–cell adhesion (e.g., higher membrane concentration of E-cadherin-*β*-catenin complexes^55^). Next, each cell was assigned a target area (*a*_0_) with changes in cell area from this value being energetically unfavourable. Area constraint has been used to model bulk cell stiffness (or inverse compressibility), and is related to the internal pressure^40^. Similarly, the perimeter constraint (*λ*_*p*_) forces the cell to maintain its perimeter close to the preferred perimeter (*p*_0_) and ensures that cells do not develop extreme deformed configurations. Hence, this constraint can be used to model the ease with which a cell can change its shape, and is therefore related to the line tension/cortical stiffness of the cell. While bulk stiffness can be estimated using Atomic Force Microscopy (AFM) experiments of cell indentation, cortical stiffness can be estimated using micropipette aspiration experiments. In our simulations, all cells have *a*_0_ = 400 *μ*m^2^ and *p*_0_ = 35 *μ*m. The last term *w*(*σ*) in Equation (1) accounts for the intrinsic motility of cells and is given by the expression 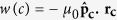
45. Here, *μ*_0_ represents the strength of cell motility, 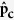 represents the unit vector in the direction of preferred polarity for cell *c* and vector **r**_**c**_ represents centre of mass of the cell *c*. While *μ*_0_ was kept constant in all simulations, the value of 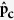 at any time *t* was modeled as an adaptive quantity set as the average of the displacements during the the previous *τ* MCS normalized to unity^45^ (Fig. 1B). Specifically, Equation (2) was used to find the preferred polarity of the cell c at time *t*, and was updated after every MCS.


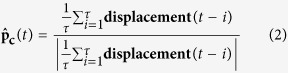


where,

*τ*: time scale of polarity evolution.

**displacement**(*t*): displacement of cell’s centre of mass *c* between *t*^th^ and (*t* + 1)^th^ MCS.

The parameter *τ* can be tuned to model persistence of cell migration. While we have used *τ* = 10 in all our simulations as done elsewhere^45^,^56^, one set of simulations were performed to study the effect of *τ* on cell invasion dynamics (Supp. Fig. S2).

### MMP dynamics and ECM degradation

In addition to the Metropolis equation based copy index events governed by Equation (1), a GGH algorithm can be integrated with other objects such as chemical field(s)^40^. The dynamics of a chemical field can be described by reaction–diffusion systems. Such chemical field can then be integrated into the main GGH algorithm by modeling the effect of this field on other system components. This approach has been used to model processes such as growth factor-dependent cell growth and oxygen-dependent cell apoptosis^43^. In our model, a separate field was maintained for soluble MMPs which are secreted by cells when they come in contact with ECM fibres (Fig. 1D). Whenever a cell comes in contact with ECM fibre(s) it secretes MMPs at the rate of *λ* per second at the site of ECM fibre(s), where *λ* represents the intrinsic MMP production rate. This ECM-dependent MMP secretion rule was chosen in line with our observations that increase in ECM density enhances MMP secretion^23^. Similar ECM density-dependent MMP secretion profile was observed when MCF-7 cells were cultured on collagen with varying densities (Supp. Fig. S1). Further, since the actual values of the MMP secretion rates are not known for cancer cells, the effect of *λ* was studied by varying its value from 0.01 to 0.1 s^−1^ 57. This secretion dynamics of MMP was modelled using CC3D ‘Secretion plugin’^58^. These secreted MMP molecules then diffuse in the medium with a diffusion coefficient (*D*) of 1.0 × 10^−9^ cm^2^.s^−1^. In addition to secretion and diffusion, soluble MMPs have a defined lifetime and degrade after some time. Therefore, MMP degradation (with a degradation rate *δ*_MMP_ of 0.002 s^−1^) was also considered in the MMP dynamics. Diffusion and degradation of MMP molecules were modelled using reaction–diffusion dynamics given by equation-(3).


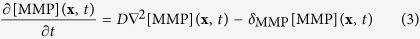


where, [MMP](**x**, *t*): MMP count at pixel **x** and time *t*

D: Diffusion coefficient of MMP

*δ*_MMP_: degradation rate of MMP

The diffusion coefficient and degradation rate were kept constant across all the simulations. Since the rate of all the MMP events (i.e. MMP secretion, MMP diffusion and MMP degradation) have units of sec^−1^, and one MCS corresponds to 36 seconds (see below), the PDE solver, used to solve the reaction–diffusion system, was called 36 times in a single MCS. Forward Euler method-based PDE solver, available as part of CC3D package, was used to solve reaction diffusion system of MMP dynamics^40^. Since one pixel in our model represents a higher order ECM structure instead of a single fibril molecule, instead of modeling the exact ECM degradation reaction, a threshold-based approach was used to model ECM degradation. Precisely, after every MCS, ECM degradation was effected at all pixel locations where MMP count was greater than or equal to *1* (Fig. 1D). A threshold of *1* was chosen as MMP secretion was also performed on a single pixel basis. A similar approach has been used to model cell quiescence and necrosis in multiscale model of cancer stem cell-driven phenotypical heterogeneity^59^. Growth factor-dependent cell growth, oxygen-dependent cell apoptosis can also be modelled using this approach^43^. At these locations, to mimic ECM degradation, ECM fibres were removed by changing the pixel type to fluid, and the count of MMP at that pixel location was reduced by 1. Removal of ECM fibres from the lattice is expected to influence cell–ECM adhesion and GGH dynamics thereby integrating MMP reaction–diffusion dynamics with GGH-based cell mechanics model.

### Parameter values and assumptions

All parameters used in the model were either chosen from the literature or determined by optimizing the model to recapitulate previously observed phenomena. Specifically, values of all adhesion energies (*J*_XX_), inverse compressibility (*λ*_*a*_) and strength of noise in the dynamics or magnitude of effective membrane fluctuations (*T*_m_) were taken directly from the literature^42^. The value of the perimeter constraint (*λ*_*p*_) was assumed in the simulations. The strength of intrinsic motility *μ*_0_ was chosen such that the cells showed rotational motion when confined within a non-degradable circular geometry^56^ (Supp. Video-V1), and showed translational motion when placed in ECM with pre-existing paths. Subsequently, the value of MCS was estimated to be 36 seconds so as to obtain cell speeds (≈23 *μ*m.h^−1^) comparable to experiments^56^,^60^ (Supp. Text). MMP diffusion timescales were estimated from the literature^61^. Since the exact life time of MMP molecules is not known, MMP degradation rate was taken from previous computational model^57^. Finally, cell–cell adhesion energy (i.e., *J*_cc_) and MMP secretion rate (*λ*) were varied to explore the effect of cell–cell adhesion and MMP secretion rate on cell migration dynamics. To probe the role of cell–cell adhesion in cell invasion, three values of *J*_cc_ (i.e., 1, 16 and 40) were chosen. These values were choosen so that (i) at the lowest value (i.e., *J*_cc_ = 1), cells have a higher propensity to adhere to other cells as compared to ECM fibres or fluid, (ii) at the moderate value (i.e., *J*_cc_ = 16), cells have equal propensity to adhere to other cells or ECM fibre and (iii) at the highest value (i.e., *J*_cc_ = 40), cells have more propensity to adhere to fluid or fibre as compared to other cells. Finally, to dissect the role of MMP secretion rate in mediating cell invasion, MMP secretion rate was varied from 0.01 s^−1^ to 0.1 s^−1^ to cover one order of magnitude as done before^57^. Although we have defined our model in generic terms by focussing mainly on cellular phenotype rather than particular cell type, the model parameters can be tuned to mimic the behavior of specific cell types. For example, high MMP secretion (*λ*) and low cell–cell adhesion (*J*_cc_ >> 1) can be used to model the behavior of highly invasive cancer cells. Similarly, low MMP secretion rate and high cell–cell adhesion can be used to mimic the behavior of less invasive cells. Other parameters, which we have kept constant in all our simulations to avoid an excess number of parameters, can be used to model other biophysical properties of the cells. For example, the fact that MDA-MB-231 cells are softer than MCF-7 cells^62^ may be modeled by using lower values of *λ*_*a*_ and *λ*_*p*_ for MDA-MB-231 cells. In our simulations we kept *λ*_*a*_ and *λ*_*p*_ constant as our intention was to probe the collective influence of cell–cell and cell–ECM interactions on cell invasion. Lastly, since the present model was aimed at studying cancer invasion only, two factors relevant to cancer progression – cell division and/or continuous supply of cells from tumour mass – were not taken into consideration in our model. Though the CC3D steppable ‘MitosisSteppable’ can be used to implement cell division^40^, not taking cell division into account helped us in reducing the parameter space of our model. In one set of preliminary simulations performed by taking cell division into account, results did not deviate much from those obtained in the absence of cell division (Supp. Section-5). Also, to study the potential role of cell supply from primary tumour on cell invasion, a set of simulations were performed where cells were continuously added to the primary cell site. While the pattern of invasion could not be compared due to very large number of cells, MMP secretion and ECM degradation profile showed simlar behaviour in the presence of cell supply (Supp. Section-6).

### Quantification of invasion

Four quantities including the total distance moved by a cell (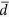), cell translocation (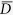), radius of gyration (RoG) and percentage ECM degradation were used to quantify cancer cell invasion. While the first three quantities were used to determine the spreading or migration capabilities of a cell, the fourth metric was used to quantify MMP-mediated ECM degradation. While 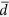 was used to track the mobility or dynamicity of a cell, 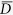 represents the displacement of a cell from its initial position and is a measure of migration persistence. In contrast to 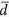 and 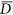 which are cell level measures, RoG was determined to calculate the population scattering. Further, since loss of basement membrane has been used as a metric of tumour invasiveness^63^, percentage ECM degradation was also quantified in our simulations to assess cancer cell invasion.

We tracked individual cell trajectories during the simulations. The population averaged total distance travelled by cells (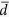) in 1800 MCS (≈18 hours) was calculated by adding the centroidal movements between successive MCS steps (i.e., 36 seconds) for each of 69 cells, and then averaging, i.e.,


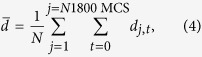


where *d*_j,*t*_ corresponds to the distance travelled by the *j*^th^ cell at MCS time step *t*, and *N* represents the total number of cells in the population (*N* = 69 in our case). In addition, the population averaged net translocation (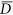) was determined by calculating the distance between the initial (*t* = 0) and final (i.e., after 1800 MCS or *t* ≈ 18 h) position of each cell, and then averaged for the whole cell population, i.e.,


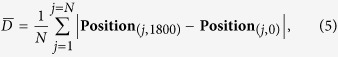


where **Position**_(j,t)_ corresponds to the position of *j*^th^ cell at MCS time *t*. To quantify the extent of cell scattering at the end of simulations (after ≈18 hours), RoG with respect to the initial population centre was also quantified as per the following expression:


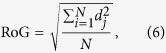


where, *N* corresponds to the total number of cells in the population and *d*_j_ is the distance of the *j*^th^ cell from the initial tumour centroid ((250, 250) in our case). Finally, ECM degradation was quantified by calculating the difference in the number of fibre pixels between the initial and final simulation steps. The percentage ECM degradation was then quantified with respect to the initial number of fibre pixels.

## Sandwich Gel Invasion Assay

For the sandwich gel invasion assay, cells were plated on collagen-coated glass-bottom dishes (HiMedia), and layered with 3D collagen I (Corning) gel on top. Coverslips were functionalized by treating with 0.1 N NaOH and silane (Sigma) for 15 minutes each followed by 0.5% glutaraldehyde (50%, Sigma) for 20 min, at room temperature. Functionalized coverslips were coated with 1 *μ*g/cm^2^ of rat tail 2D collagen I (C3867, Sigma). MCF-7 cells were plated on the coverslips in DMEM media (Gibco) supplemented with 10% FBS (HiMedia) and allowed to incubate for 3 hours in the CO_2_ incubator. Cells were plated at two specific densities (5 × 10^3^ and 25 × 10^3^ cells per cm^2^) so as to have a predominance of single cells at the lower density and groups of cells at the higher density. Post incubation, media was aspirated and cells were layered with 3D collagen type I on top at three different densities of 0.5, 1 and 2 mg/ml, respectively, as per previously established protocols^64^. 3D collagen was allowed to gel by keeping the entire set up at 37 °C in the CO_2_ incubator for 30 minutes. Post gel formation, fresh media was added on top and kept for incubation for 24 hours. For protease inhibition drug studies, fresh media was supplemented with 5 *μ*M of GM6001 drug (Merck). Imaging was done with an inverted phase contrast microscope (Olympus) at 20× magnification over a period of 12 hours at 20 minutes interval. Total cell translocation (distance moved from initial position) was calculated after 12 hours using the manual tracking plugin of ImageJ (NIH). For single cell migration, trajectories of individual cells were tracked. For collective cell migration, trajectories of cells moving in groups were quantified to determine the net translocation. Atleast 20 cells per condition were analyzed and the experiments repeated twice.

## Results

### Collective cell invasion requires lesser ECM degradation than single cell invasion

**Figure 2:**
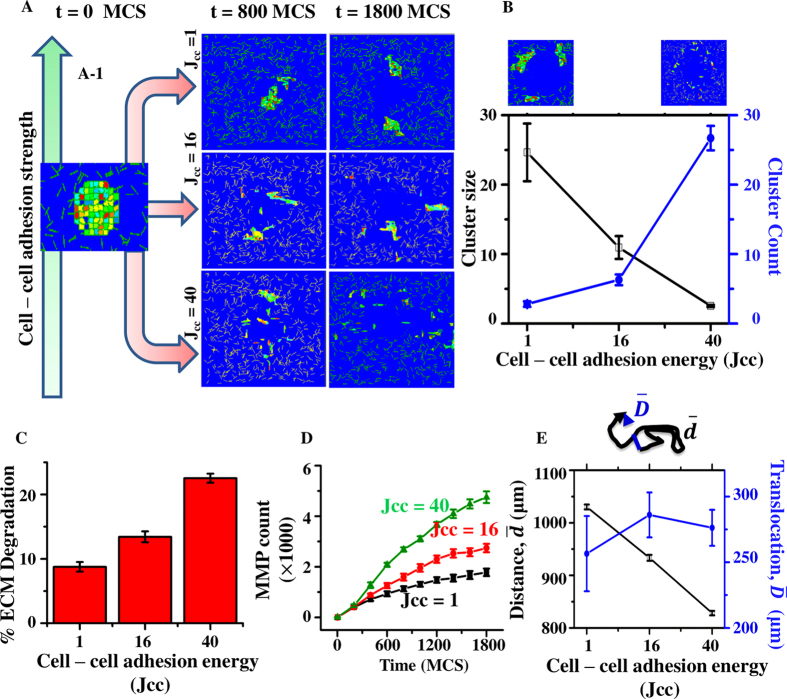
Influence of cell–cell adhesion energy (*J*_cc_) on invasion efficiency. (**A**) Temporal patterns of invasion of a group of cells enmeshed in a fibrillar ECM network for different levels of cell–cell adhesion. **Upper Panel:** High cell–cell adhesion (*J*_cc=1_). **Middle Panel:** Medium cell–cell adhesion (*J*_cc_ = 16). **Lower Panel**: Low cell–cell adhesion (*J*_cc_ = 40). **Model components:** Blue pixels represent fluid, yellow pixels represent ECM fibres and coloured boxes represent cells. (**B**) Quantification of cell migration pattern with number of clusters and average size of cell cluster after 1800 MCS. (**C**) percentage ECM degradation for different values of *J*_cc_. (**D**) Temporal profile of total number of MMP molecules present in the lattice for varying values of *J*_cc_. (**E**) Total distance moved by cells (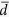) and net cell translocation (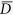) for different values of *J*_cc_. Inset shows representative trajectory of a single cell. Error bars: ±standard error of mean (SEM).

Cell-cell adhesions represent one of the key factors governing cancer metastasis. While loss of cell–cell adhesion is known to trigger EMT^65^,^66^,^67^,^68^,^69^, overexpression of cell–cell adhesion checks EMT^70^,^71^,^72^. In contrast to the single cell mode of invasion where cell–cell adhesion does not play any role, many cancer cells are also known to exhibit collective invasion wherein cell–cell adhesions remain intact^73^. To see if our model can recapitulate the influence of cell–cell adhesion on the mode of invasion, we performed simulations for varying values of cell–cell adhesion energy (*J*_cc_), that is *J*_cc_ = {1, 16 and 40}, with *J*_cc_ = 1 corresponding to high cell–cell adhesion, *J*_cc_ = 16 corresponding to medium cell–cell adhesion, and *J*_cc_ = 40 corresponding to low cell–cell adhesion. In these simulations, MMP secretion rate (*λ*) was kept constant at *λ* = 0.05 *s*^−1^. All other parameters were kept fixed (as described in Table 1). For these simulations, a cell aggregate of 69 cells of initial diameter 20 *μ*m each were placed at the centre of a randomly aligned ECM lattice (Fig. 2A left-most). The ECM lattice was generated using a custom written C++ utility (Supp. Text). For comparing the cell invasion patterns obtained for different values of *J*_cc_, simulations were run for 1800 MCS (≈18 hours), and repeated 10 times per condition for statistical comparison between the different cases.

The *J*_cc_ values chosen to mimic different extents of cell–cell adhesion successfully recapitulated the different modes of invasion (Fig. 2A, Supp. Videos V2,V3–V4). While cells were found to scatter in different directions when cell–cell adhesion was low (i.e., *J*_cc_ = 40) (Fig. 2A right-lower panel), cells moved collectively when cell–cell adhesion was high (i.e., *J*_cc_ = 1) (Fig. 2A right-upper panel). To get a quantitative estimate of the mode of cell invasion for varying cell–cell adhesion, clustering of cell population was quantified by counting the number of clusters and the size distribution of the clusters at the end of the simulation (i.e., after 1800 MCS). While the population having larger cluster size and lower number of clusters resembles the collective mode of migration, the population with smaller cluster size and higher number of small clusters is indicative of individual mode of migration. The quantification showed that the loss of cell–cell adhesion, i.e., increase in *J*_cc_ values, triggered the breaking of the population into large number of smaller clusters, thereby leading to the individual cell invasion (Fig. 2B). These results successfully recapitulate the observed EMT process where loss of cell–cell adhesion triggers mesenchymal model of migration^65^,^66^,^67^,^68^.

Since cell invasion requires ECM degradation, the simulation data was analyzed to determine the extent of ECM degradation for different extents of *J*_cc_. This was done by quantifying the percentage reduction in the total number of fibre pixels at the end of 1800 MCS. Surprisingly, in spite of having identical MMP secretion rate (i.e., *λ* = 0.05 s^−1^) and no direct dependence of fibre degradation on cell–cell adhesion, the population of cells with differing cell–cell adhesion propensities showed significant difference in ECM degradation, with minimum degradation observed at highest cell–cell adhesion and maximum degradation observed at lowest cell–cell adhesion (Fig. 2C). Since fibre degradation is mediated by MMP molecules, to check the basis for lesser degradation at higher cell–cell adhesion, the temporal profile of total MMP count was tracked for different values of cell–cell adhesion. Interestingly, though all cells possessed the same MMP secretion capabilities, MMP secretion was highest at lowest cell–cell adhesion (Fig. 2D) indicating that the lower degradation at higher cell–cell adhesion was due to decrease in levels of MMP secretion. This decrease in MMP density for population of cells with high cell–cell adhesion may be due to lower cell–ECM interaction, i.e., the number of ECM fibres in contact with single cells is greater than when the cells are in clusters, leading to greater MMP secretion by single cells than cells in groups.

Given that cell scattering leads to greater ECM degradation, we next tested if this enhanced degradation led to enhanced invasion. Invasion was characterized by two different metrics: the total distance moved by the cells (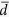) and the net cell translocation (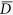) (i.e., displacement of cells from their respective initial positions to their final positions) after 1800 MCS. While greater 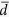 implies greater movement, it may not imply greater invasion, as it is possible for a cell to return to it’s starting point, particularly if it moves diffusively. Hence, 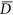 was tracked as a metric of effective invasion. The total distance travelled by cells (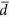) was maximum when cells moved collectively, and dropped sharply with decrease in cell–cell adhesion (Fig. 2E). Greater distance moved by cells in groups was due to dynamic rearrangements within the group wherein cells constantly changed their positions within the cluster as they moved collectively (Supp. Video V2). However, single cells moved by a lesser distance compared to cells in groups suggestive of more persistent invasion by single cells, achieved through higher ECM degradation (Fig. 2E)^74^. Compared to 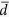, the net cell translocation (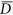) was less sensitive to changes in cell–cell adhesion, with minimum value observed at highest cell–cell adhesion (Fig. 2E). The net cell translocation was also dependent on the time scale of polarity evolution (*τ*), with increase in *τ* values leading to increase in cell translocation for all values of cell–cell adhesion (Supp. Fig. S2, materials and methods). In addition to 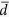 and 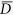, RoG, another indicator of cell scattering, was also quantified and found to have cell–cell adhesion dependency similar to 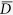 (Supp. Fig. S3A). Additionally, the big error bars in 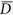 and RoG measures arise due to the heterogeneity in the distribution of cell translocation (Supp. Fig. S3B). Together, our results suggest that while loss of cell–cell adhesion leads to individual mode of migration, our model predicts that the collective invasion requires lesser ECM degradation than single cell invasion, with dynamic re-arrangements occurring in groups of collectively moving cells.

### MMP secretion rate influences cell invasiveness and invasion pattern

**Figure 3:**
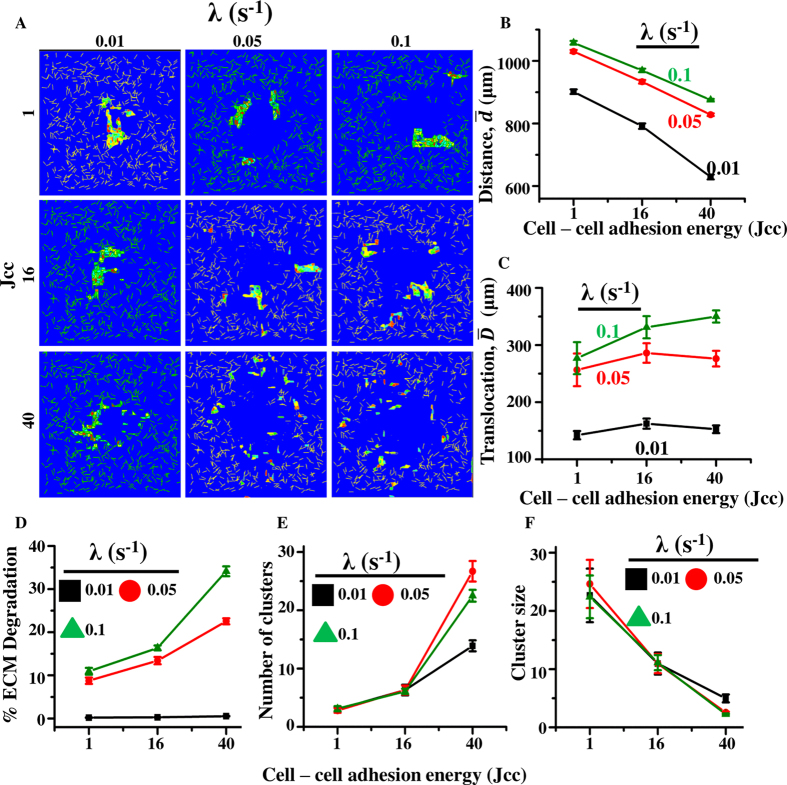
Collective influence of MMP secretion rate (*λ*) and cell–cell adhesion (*J*_cc_) on cancer cell invasiveness and migration pattern. (**A**) Represented images of cell migration pattern for varying cell–cell adhesion (*J*_cc_) and for varying MMP secretion rates (*λ*) after 1800 MCS. (**B**) Total distance moved by cells for varying λ and *J*_cc_. (**C**) Net translocation of cells from their respective initial positions. (**D**) percentage ECM degradation at different MMP secretion rates and cell–cell adhesion. (**E**,**F**) Mode of invasion quantified by tracking number of clusters (**E**) and cluster size (**F**) after 1800 MCS. Error bars: ±SEM.

Though no direct inter-dependence between cell–cell adhesion and MMP secretion rate was assumed, our simulation results demonstrate that lesser degradation is achieved when cells move collectively. While the above simulations were performed for a fixed MMP secretion rate (λ = 0.05 *s*^−1^), given that different cell types possess different proteolytic abilities, it is possible that MMP secretion rate may dictate the pattern of invasion. To test this, simulations were performed for varying cell–cell adhesion (*J*_cc_ = 1, 16 and 40) and varying MMP secretion rates (λ = 0.01–0.1 s^−1^). As before, quantification was done after 1800 MCS. As expected, increase in MMP secretion rate led to increase in both cell movement and cell translocation for all values of *J*_cc_ (Fig. 3A–C), with greatest amplification at lowest cell–cell adhesion (i.e., *J*_cc_ = 40). A similar influence was observed for cell scattering quantified using RoG of the cell population (Supp. Fig. S4). This enhancement in invasion at higher MMP secretion rates was associated with greater ECM degradation (Fig. 3D). Further, quantification of cluster size and number of clusters revealed differences only when cell–cell adhesion was weak (i.e., *J*_cc_ = 40), with increase in MMP secretion rate leading to further scattering indicative of single cell invasion (Fig. 3E,F). Together, these results suggest that increase in MMP secretion rate leads to increased invasion and ECM degradation^23^.

### Increase in MMP secretion rate increases efficiency of collective invasion in dense matrices

**Figure 4:**
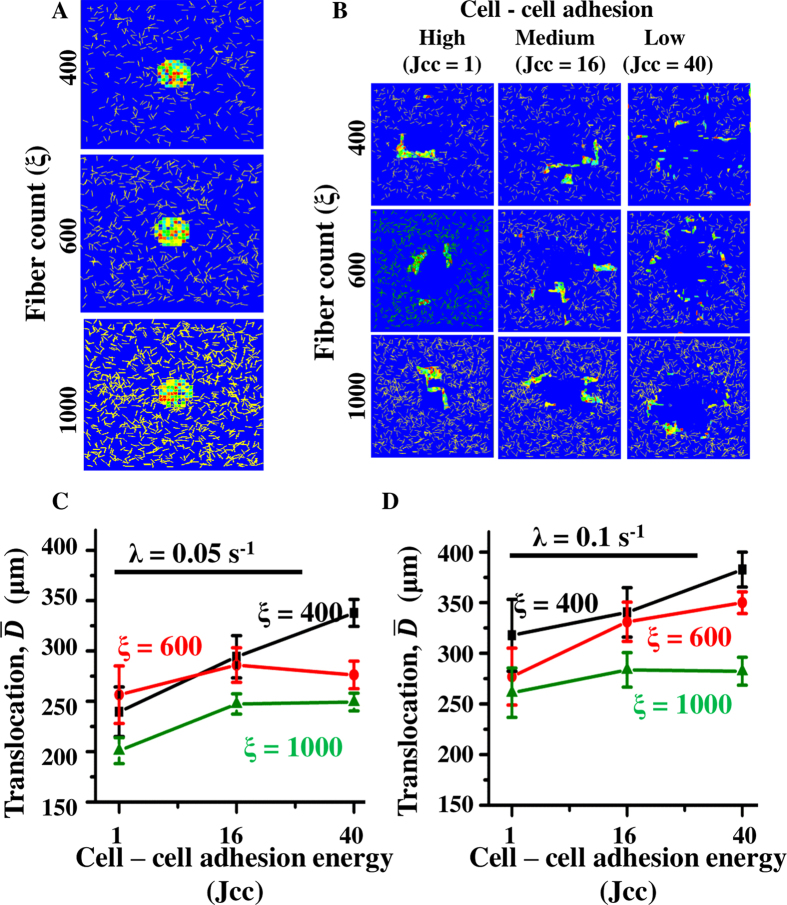
Combined influence of MMP secretion rate and ECM density on invasion. (**A**) Position of cell cluster at the beginning of simulation for three different fibre densities (*ξ* = 400, 600 and 1000, respectively). (**B**) Pattern of cell scattering at the end of 1800 MCS at varying fibre densities and varying *J*_cc_. MMP secretion rate was kept constant at *λ* = 0.05 s^−1^. (**C**) Net translocation of cells at varying fibre densities and varying *J*_cc_. (**D**) Net translocation of cells at varying fibre densities and varying *J*_cc_ for *λ* = 0.1 s^−1^. Error bar: ±SEM.

In the simulations thus far, fibre density was kept constant. However, density of the tumour-associated ECM has been shown to play a pivotal role on cancer progression^13^,^23^,^24^,^25^,^65^,^75^, with increased deposition of fibrillar ECM proteins like collagen and fibronectin reported in various epithelial cancers^16^,^76^,^77^. Such an increase in ECM density has been shown to cause increased ECM degradation via increase in MMP secretion rates^23^. Increased ECM density has also been shown to foster collective cell migration through cell jamming^78^. However, the combined influence of cell–cell adhesion and ECM density on cell invasiveness is incompletely understood. To address this question, simulations were performed at varying fibre densities for varying extents of cell–cell adhesion. MMP secretion rate was kept constant at *λ* = 0.05 sec^−1^. ECM fibre density was varied by varying the number of ECM fibres (*ξ*) in the lattice (*ξ* = 400, 600 and 1000, respectively) (Fig. 4A). Simulation results suggested that the invasion dynamics was significantly altered with change in fibre density, with increase in ECM density limiting the extent of invasion (Fig. 4B). Given that in our simulations, the total number of MMPs secreted is proportional to the number of ECM fibre pixels in contact with the cells (Materials and Methods), higher fibre densities should lead to greater MMP secretion and higher ECM degradation. While this was found to be true (Supp. Fig. S5A, S5B), the net translocation was still minimum at the highest fibril density (i.e., *ξ* = 1000), demonstrating the dominant influence of ECM density in limiting invasion (Fig. 4C). Similar to 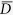, the RoG-based assessment of population scattering also suggested that the cell scattering was suppressed greatly at the highest fibre density (i.e., *ξ* = 1000) (Supp. Fig. S5C). Moreover, increase in cell–cell adhesion further amplified the effect of fibre density, with higher cell–cell adhesion leading to greater reduction in the extent of invasion.

In our model, in addition to fibre density, MMP secretion also depends on the MMP secretion rate (*λ*). Since fibre density-mediated MMP secretion alone was not able to circumvent the effect of steric hindrance on cancer invasion, we next investigated if increase in MMP secretion rate can promote cancer invasiveness. To test this, simulations were performed with a two-fold higher value of MMP secretion rate (i.e., *λ* = 0.1 sec^−1^ (Supp. Fig. S5D). Increase in MMP secretion rate led to increase in the extent of ECM proteolysis (Supp. Fig. S5E) and translocation across all the conditions, with greatest increase observed when cell–cell adhesion was high (Fig. 4D). Together, these results suggest that while increase in ECM density hinders cell invasion, increase in MMP secretion rate fosters it. Interestingly, it was observed that while two fold increase in MMP secretion rate was sufficient to suppress the effect of increase in ECM density (from *ξ* = 400 to *ξ* = 1000) at moderate and high cell–cell adhesion (i.e., *J*_cc_ ≤ 16), only partial suppression was achieved at low cell–cell adhesion (i.e., *J*_cc_ = 40). Cell scattering, quantified using RoG, was also increased with increase in MMP secretion rate and showed similar dependence on cell–cell adhesion (Supp. Fig. S5F). Together, these results indicate that when cells invade collectively through dense matrices, increasing the MMP secretion rate may be one of the strategies to increase invasion efficiency. Conversely, if the MMP secretion of the cells is on the lower side, cells can invade more effectively if they migrate individually by undergoing EMT.

### Alignment of ECM fibres can sustain invasion without proteolysis

**Figure 5:**
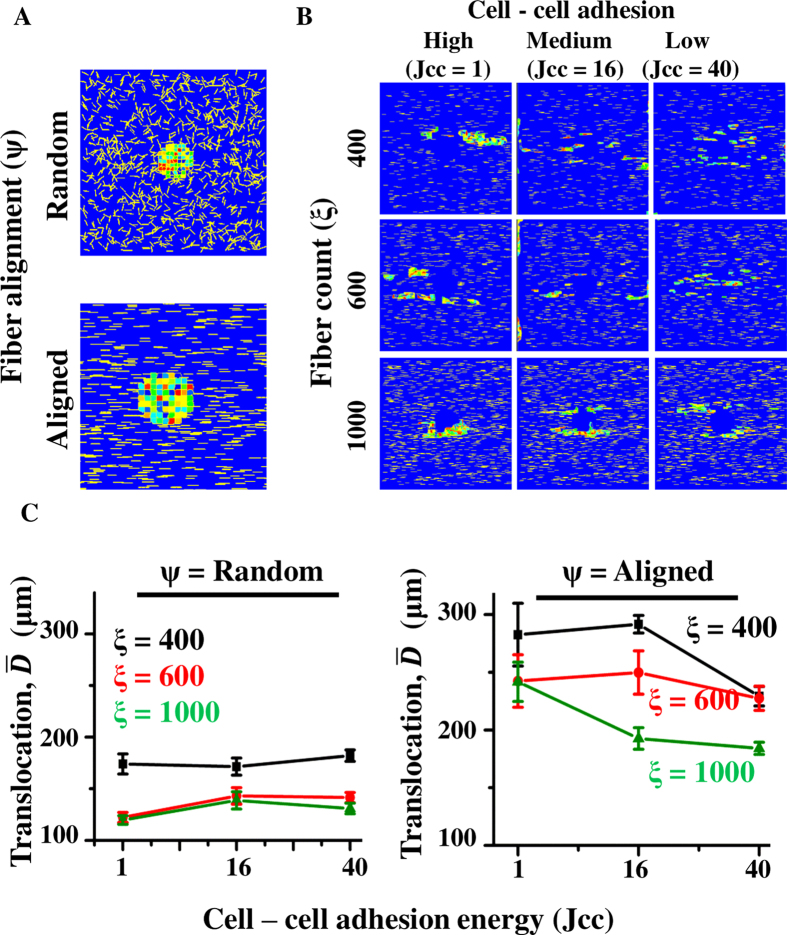
Aligned matrices can sustain cell invasion even without ECM proteolysis. (**A**) Position of cell cluster at the beginning of simulations for two different fibre orientations (*ψ*) (random and aligned) (*ξ* = 1000). (**B**) Representative images for cell migration pattern for aligned matrices at varying ECM densities and cell–cell adhesion. MMP secretion was turned off. (**C**) Net translocation of cells at varying fibre densities and varying *J*_cc_ in random and aligned matrices. Error bar: ±SEM.

In addition to alterations in ECM density, ECM organization also undergoes significant alterations during cancer progression^15^. This is brought about by enzymes like lysyl oxidase (LOX) which crosslink the matrix and make it more linearized^20^. Such alignment of matrix has been shown to trigger EMT and increase cancer invasiveness^63^. Simulations performed with aligned ECMs at the highest density (i.e., *ξ* = 1000, to mimic stiff matrices) in the presence of proteolysis showed that loss of cell–cell adhesion indeed increased cell translocation and ECM degradation (Supp. Fig. S6). These results are consistent with recent findings where induction of EMT (which causes loss of cell–cell adhesion)^79^ and ECM stiffness were shown to increase tumour invasiveness^63^. While secreted MMPs degrade tumour ECM, thereby making tracks for migration, crosslinking of fibres may enable cells to make use of aligned fibres as a contact guidance cue and migrate with minimum ECM proteolysis utilizing the space available between neighboring sets of crosslinked fibres. To test the hypothesis that matrix alignment can sustain cell migration by providing contact guidance, simulations were performed without proteolysis at different fibre densities and two fibre orientation(*ψ*): random and aligned (Fig. 5A). To generate aligned matrices, C++ routine was used to generate a mesh of fibres with all fibres aligned in the horizontal direction (Supp. Text). The generated *.PIFF files were then used as input in the CompuCell3D model. In these simulations, in addition to varying fibre density and orientation, cell–cell adhesion was also varied to test how single cell invasion and collective cell invasion fared under these conditions. In the absence of proteolysis, in matrices with randomly aligned fibres, cell translocation was limited to the same extent for all values of cell–cell adhesion, with highest translocation observed at the lowest fibre density (Fig. 5B,C-left, Supp. Videos V5,V6,V7,–V8). Translocation was significantly enhanced when fibres were aligned, with maximum translocation observed at lowest fibre density and highest cell–cell adhesion (Fig. 5C, Supp. Fig. S7A, Supp. Videos V9,V10,V11–V12). Consistent with greater translocation in aligned matrices, cell scattering also increased in aligned matrices (Supp. Fig. S7B). In addition to increase in invasiveness, contact guidance was quite observable in aligned matrices where cells moved in the direction of fibre alignment (Supp. Videos V9,V10,V11–V12). Our model results predict that ECM fibre alignment can sustain both singly invading cells and the cells invading in groups. To assess the role of cell–ECM adhesion in sustaining this invasion, simulations were performed where cell–ECM adhesion energy (i.e., *J*_ce_) was set equal to cell–fluid interface energy (i.e., *J*_cf_) to model a case where cells had no preference to adhere to an ECM fibre. On quantification, except for a mild increase (specifically for *ξ* = 1000, *J*_cc_ = 1), no major changes in cell translocation were observed upon decreasing the cell–ECM adhesion (Supp. Fig. S8). Together, our results suggest that ECM alignment is capable of sustaining both single cell and collective cell invasion even in the absence of ECM proteolysis.

### Comparing model results with experiments: invasion of MCF-7 cells using sandwich gels

**Figure 6:**
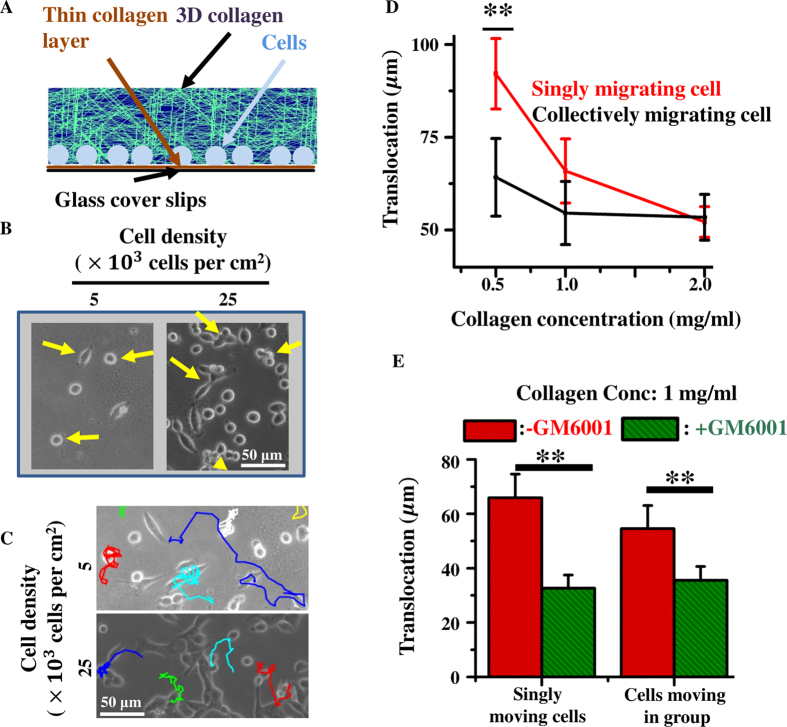
Invasion of MCF-7 cells in sandwich cultures. (**A**) Cells were cultured on collagen-coated glass coverslips and overlaid with a layer of 3D collagen so as to create a sandwich. (**B**) Representative images of cell positioning at *t* = 0 at two different cell densities. (**C**) Tracks of cell migration for singly invading cells and cells invading in groups. (**D**) Translocation of MCF-7 cells moving singly and in groups. (**E**) Translocation of MCF-7 cells in sandwich gels fabricated with 1 mg/ml collagen in the presence of the broad spectrum MMP inhibitor GM6001. Errorbar: ±SEM. ‘**’ indicates statistical significance (*p* < 0.05).

Our *in-silico* results have demonstrated the contributions of cell–cell adhesion (*J*_cc_), MMP secretion rate (*λ*), ECM density (*ξ*) and ECM alignment (*ψ*) in regulating tumour cell invasion. In these simulations, to maintain generality of cell invasion, we focused mainly on variation in cellular phenotype (*J*_cc_ and *λ*) and ECM properties (*ξ* and *ψ*) without focusing on specific cell line. To compare and contrast our simulation results with a particular cell line, we performed invasion studies with MCF-7 cells using sandwich gels. Similar to our computational framework wherein cells invade outward into the surrounding matrix, for the experimental studies, MCF-7 cells were plated on collagen coated glass coverslips and overlayed with 3D collagen (Fig. 6A) so as to create a sandwich. To study the influence of ECM fibre density on cell invasion, three densities of 3D collagen (0.5, 1.0 and 2.0 mg/ml) were used. Cell invasion was studied using MCF-7 cells plated at two densities (5 × 10^3^ and 25 × 10^3^ cells per cm^2^) to study invasion by both single cells and cells moving in groups (Fig. 6B, Supp. Videos V13 and V14) (Materials and methods). Cell invasion was tracked for 12 hours and cell translocation (displacement from initial position) was determined using the ‘Manual Tracking’ plug-in of ImageJ. For single cell migration, trajectories of individual cells were tracked. For collective cell migration, trajectories of cells moving in groups were quantified (Fig. 6C).

Consistent with our simulation results (Fig. 4), increase in collagen density caused a drop in cell translocation for singly invading cells. However, almost no difference was observed for collectively invading cells (Fig. 6D). Specifically, for singly invading cells, increase in collagen density from 0.5 mg/ml to 1 mg/ml caused a significant drop (~50%) in cell translocation. A similar trend was observed in our simulations for *λ* = 0.05 and 0.1 s^−1^ and *J*_cc_ = 40 (low cell–cell adhesion) when ECM fibre density (*ξ*) was increased from 400 to ≥600 (Fig. 4C,D). When experiments were performed with the MMP inhibiting drug GM6001 on 1 mg/ml collagen gels, in line with our simulation results (Fig. 5C-Left), a drop in cell translocation was observed in both singly moving and collectively moving cells, highlighting the direct contribution of MMPs in sustaining invasion (Fig. 6E). Similar to our results, GM6001 treatment has also been shown to inhibit the invasion of MDA-MB-231 cells from 3D spheroids embedded in 3D native type 1 collagen gels^81^. In conclusion, in line with our simulation predictions, our experimental results demonstrate the influence of ECM density in providing steric hindrance to cell movement and the role of MMPs in reducing this hindrance by proteolysis.

## Discussion

**Figure 7:**
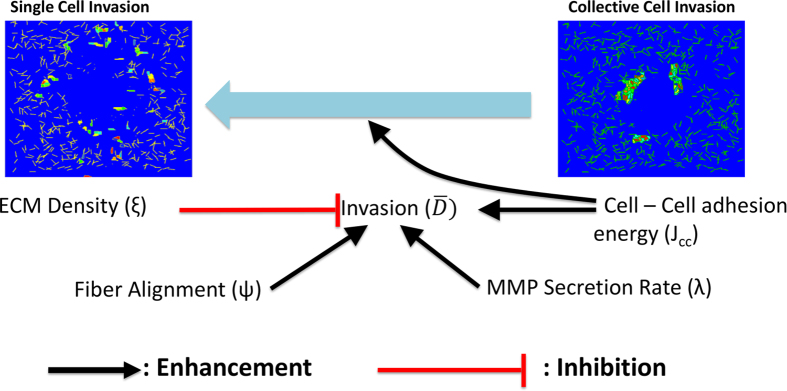
Regulation of cell invasiveness and mode of invasion. Cancer cells can switch between different strategies to invade. Collective invasion is the more efficient form of invasion since it requires lesser overall secretion of MMP and ECM fibril degradation, when compared to individual cell invasion. Increased ECM density for randomly aligned fibres provide greater confinement for the migrating cells and reduce their invasion. However, the cells can increase their MMP secretion rate to counter this effect and still invade collectively. If the cells cannot secrete enough MMP, then they can undergo EMT by loss of cell–cell adhesions (i.e., increasing cell–cell adhesion energy, *J*_cc_) and invade individually. If the cells have lower MMP secretion rate, then they can align the ECM fibrils for effective invasion, irrespective of cell–cell interaction energies.

Cell invasion is central to cancer metastasis with ECM properties regulating the efficiency and pattern of invasion. In this paper, we present an *in-silico* model to understand the modes of cancer cell dissemination from a group of cells. Our study demonstrates the importance of ECM density and organization in fostering collective invasion through both proteolytic and non-proteolytic mechanisms. Our model makes two important predictions. First, our model predicts that collective cell invasion requires lesser ECM degradation compared to individual cells for achieving similar invasion, suggesting collective cell invasion may be more efficient than single cell invasion. While increase in ECM density inhibits collective cell invasion, higher MMP secretion rate is required for sustaining it, particularly in dense matrices. Additionally, although increase in MMP secretion enhances both single and collective cell invasion, inhibition of MMP secretion in individually migrating cells causes a transition to collective cell invasion as observed experimentally^78^. Secondly, our results suggest that both single and collective cell invasion can be sustained without any proteolysis in aligned matrices. In line with our *in-silico* predictions, our experiments demonstrate the influence of ECM density in restricting the invasion of MCF-7 cells by providing steric hindrance and the necessity of MMP activity for sustaining invasion. Collectively, our study highlights the importance of ECM density and organization in dictating cell invasiveness, and illustrates the multiple mechanisms for collective invasion to be sustained (Fig. 7).

Although both single and collective cell invasion have been observed during cancer progression, a direct comparison between the two modes of invasion has not been done due to experimental challenges. Further, since both modes of invasion require cells to move by degrading the surrounding ECM, how ECM degradation is altered between single and collective cell invasion remains unclear. Our results suggest that, at moderate to high ECM densities, collective invasion is more efficient compared to single cell invasion as cells moving in groups invade to the same extent with lesser ECM degradation (Fig. 2C,E). This was also observed experimentally at collagen densities of 1 mg/ml and higher. Spheroid invasion experiments in 3D gels often show groups of cells invading radially outward with few isolated individual cells invading outward faster. Given the higher degradation potential of single cells, it is possible that these single cells serve as ‘leader cells’ and increase the efficiency of collective invasion by reducing steric hindrance and creating small migration tracks that can be subsequently widened by collectively moving cells with lesser degradation^11^. Alternatively, it is also possible that collective cell invasion may be possible without any leader cells. This is clear from our simulations wherein collective invasion was unaltered even though cells continuously switched places within the group (Supp. Video V2). Though such motion of cells within the group during invasion is yet to be experimentally demonstrated, epithelial cells are known to exhibit rotational motion under confinement both *in vitro* and *in vivo*^81^. This rotational motion is attributed to our polarization evolution rule, which tends to align the polarization of any cell with its velocity and allows for cells to continue to move even in confined geometries^56^ (Supp. Video V1). The continuous movement of cells moving collectively within the group also implies that for achieving invasion to the same extent as that of single cells, collectively moving cells take a more circuitous route, therefore suggesting that collective cell invasion is less persistent than single cell invasion.

Higher MMP secretion is typically associated with enhanced ECM degradation^11^,^23^,^57^,^82^. Though basal value of MMP secretion rate is a cell type-specific property, the absolute number of MMPs secreted by a given cell is sensitive to several intrinsic and extrinsic factors. While integrin-mediated cell–ECM signalling can increase MMP activity^83^, MMP secretion can be inhibited or abolished pharmacologically using protease inhibitory drugs like GM6001 and SB3-CT^11^,^23^. Though increase in MMP secretion rate increased the efficiency of both single and collective cell invasion, our results suggest that single cell invasion at low to intermediate ECM density is more sensitive to MMP secretion rate (Fig. 3C,D). This may be due to greater contact of single cells with the surrounding ECM as opposed to collectively moving cells, where only the outer layer of cells are in contact with the matrix. Higher invasive potential of cells upon EMT may also be attributed to greater cell–ECM interactions leading to greater ECM degradation^84^,^85^. Together, these results highlight the sensitivity of single cell invasion to MMP secretion rate.

*In vivo*, the collagen-rich interstitial matrix is known to inhibit cell invasion. Paradoxically, cancer progression is associated with increased deposition of fibrillar collagen, with increase in ECM density correlated with increased malignancy. Increased invasiveness of cancer cells in dense matrices may be driven by enhanced integrin signalling, which in turn activates MMP-mediated ECM degradation^86^,^87^,^88^,^89^,^90^. Thus, cancer invasiveness depends both on ECM density and levels of MMP secretion. To study their collective influence on invasion, simulations were performed at different ECM fibre densities and moderate levels of MMP secretion. Our results demonstrate that increase in ECM density suppresses single cell invasion to a greater extent. In contrast, collective cell invasion is less sensitive to changes in ECM density (Fig. 4B,C). Similar to our simulation results, invasion of MCF-7 cells on sandwich collagen gels of varying densities also showed a significant decrease in cell translocation for individually moving cells and almost no change in cell translocation for collectively moving cells (Fig. 6D). Additionally, our simulation results also illustrate that increase in MMP secretion rate can mitigate the effect of increase in ECM density thereby fostering invasion. For example, the suppression in collective invasion effected by an increase in ECM density from *ξ* = 400 to *ξ* = 1000 is completely abolished by a 2-fold increase in MMP secretion rate (Fig. 4D). Such increase in MMP secretion can be induced by ECM density itself, as observed in experiments of MDA-MB-231 cells cultured on substrates of varying collagen density^23^. However, for single cells, the two-fold increase in MMP secretion rate only partially overrides the increase in steric hindrance effected by increase in ECM density. Thus, these results suggest that invasiveness is collectively regulated by ECM density and MMP secretion rate, and cells can switch between single and collective mode of invasion depending on the levels of MMP secretion in order to invade effectively.

In the presence of MMPs, loss of cell–cell adhesion and alignment of ECM fibres have been shown to increase cell invasion^63^. Consistent with these observations, our simulations for aligned and dense matrices showed greater ECM invasion for cells with low cell–cell adhesion (Supp. Fig. S6). In contrast to MMP-mediated proteolytic invasion, inhibition of MMPs induces cells to opt for a non-proteolytic mechanism of cell migration, wherein cells use actomyosin contractility to squeeze through pre-existing pores in the matrix^91^. It is possible that the efficiency of such non-proteolytic modes of invasion can be further enhanced by alignment of ECM fibres, which serve as contact guidance cues. Such linearization of matrices has been documented *in vivo* via crosslinking of collagen fibres through enzymes like LOX^20^. However, the extent to which MMPs may be necessary for this mode of invasion is unclear. Our simulations of invasion in aligned matrices without any MMP activity suggest that aligned matrices can provide suitable contact guidance cues that can sustain invasion even without any proteolysis (Fig. 5). Interestingly, our results also suggest that collectively migrating cells can maximally use the contact guidance provided by aligned ECM fibres as ECM alignment induced faster invasion at high cell–cell adhesion.

Although our model was used for studying cell invasion in the context of carcinomas, this model can easily be extended to understand invasion in other types of cancers. First, many of the components of our model are also applicable for other types of cancers. For example, cell–ECM adhesion plays a critical role in invasion in other types of cancers as well including, gliomas and sarcomas^92^. Thus, invasion of glioma cells embedded as spheroids in collagen gels, can also be explored using multiscale CPM computational models^93^. Further, the case of *J*_cc_ = 40 which models single cell invasion is applicable for all cells invading individually, and is hence applicable to nearly all types of cancers^91^,^94^. Further, this model can easily be extended to study the influence of other physiological structures that play a part in cancer invasion. For example, similar to tissue specific cells modeled in^42^, one can easily incorporate non-degradable tissue specific structures (e.g., cells or other interfaces) to model tracks such as subpial glial space and vessel walls which are used by invading cells to invade the brain^94^.

In summary, this paper demonstrates the multiple ways in which collective cell invasion can be sustained by tuning several cellular and sub-cellular processes and ECM features. However, no cellular heterogeneity within the tumour population was assumed in these simulations. Given the existence of cellular heterogeneity within the tumour population^95^, understanding the effect of ECM density and organization on cancer invasion of a heterogeneous population will be one of the future directions of this work.

## Additional Information

**How to cite this article**: Kumar, S. *et al.* Proteolytic and non-proteolytic regulation of collective cell invasion: tuning by ECM density and organization. *Sci. Rep.* **6**, 19905; doi: 10.1038/srep19905 (2016).

## Supplementary Material

Supplementary Information

Supplementary Video 1

Supplementary Video 2

Supplementary Video 3

Supplementary Video 4

Supplementary Video 5

Supplementary Video 6

Supplementary Video 7

Supplementary Video 8

Supplementary Video 9

Supplementary Video 10

Supplementary Video 11

Supplementary Video 12

Supplementary Video 13

Supplementary Video 14

## Figures and Tables

**Table 1:** Values of parameters used in simulations.

Parameter	Value	Remark
Lattice size	1 × 1 mm^2^ (500 × 500 pixels^2^)	Assumed
Initial population size (diameter)	200 *μ*m	Assumed
Target cell size	400 *μ*m^2^	Typical cell size
Fiber count (ξ)	400, 600 and 1000	Varied
Fiber orientation (ψ)	Random and aligned	Varied.
Adhesion energies	(*J*_cc_, *J*_ce_, *J*_cf_, *J*_ef_, *J*_ee_, *J*_ff_) = (1–40^*^, 16, 32, 35, 5, 35) *k*_*B*_*T*/*L*	42, *: *J*c*c* = 1, 16 and 40 were used to model different extents of cell–cell adhesion.
Area constraint (*λ*_*a*_)	1.0 *k*_*B*_*T*/*L*^4^	42
Noise strength in the dynamics (or effective membrane fluctuations) (*T*_m_)	0.01 *k*_*B*_*T*	42
Perimeter constraint (*λ*_*p*_)	0.5 *k*_*B*_*T*/*L*^2^	Assumed
Strength of motility (*μ*_0_)	50 *k*_*B*_*T*/*L*	Determined, Supp. Text
Simulation step (MCS)	36 seconds	Determined, Supp. Text
MMP Diffusion coefficient (*D*)	1.0 × 10^−9^ cm^2^.s^−1^	61
MMP Degradation rate (*δ*_MMP_)	2 × 10^−3^ s^−1^	Based on previous computational study^57^
MMP Secretion rate (*λ*)	0.01–0.1 s^−1^	Varied.

*L* is the dimension of the pixel (2 *μ*m) over which Monte Carlo moves are performed.
