# Supplementary material for: Proteolytic and non-proteolytic regulation of collective cell invasion: tuning by ECM density and organization
Source: Sci Rep. 2016 Feb 2;6:19905. doi: 10.1038/srep19905 (PMC4735823; doi:10.1038/srep19905)
Supplement: Supplementary Information [file srep19905-s1.pdf]

Supplementary Text for: Proteolytic and non-proteolytic regulation of collective cell invasion: tuning by ECM density and organization

Sandeep Kumar[1], Aastha Kapoor[1], Sejal Desai[1], Mandar M inamdar[2,*], and Shamik Sen[1,*]

[1]Department of Biosciences and Bioengineering, , IIT Bombay, Mumbai, India
[2]Civil Engineering Department, IIT Bombay, Mumbai, India
[*]minamdar@iitb.ac.in, shamiks@iitb.ac.in

1 Pilot simulations for calibrating values of perimeter constraint (λ_p) and intrinsic motility (μ_0)

The value of perimeter constraint (λ_p) was assumed in the model, and a value of 0.5 was chosen after several simulations so as to minimize excessive cell deformations (data not shown). This value of λ_p was then used for calibrating the value of cell motility strength μ_0. The strength of intrinsic motility μ_0 was optimized such that (i) the cells showed a rotational motion, similar to,[1] when confined within a non-degradable circular geometry, and, (ii) the cells showed a translational motion when placed in an extracellular matrix with already available free path. Similar to the observations of,[1] cells, when placed inside a non-degradable confinement, exhibit rotational motion (Supp. Video V1). After running several simulations, a value of $\mu_0 = 50$ was determined to satisfy the above constraints. To determine the approximate value of 1 MCS in our simulations, we run the simulations in hindrance free environment (i.e., without ECM fibres) and calculated the cell speed in units of μm.MCS^{-1}. This value of speed ($\frac{425}{1800} \approx 0.23$ μm.MCS^{-1}) was then equated to the experimentally reported values of cell speed (≈ 23 μm.h^{-1})[2] to estimate the value of 1 MCS. From this, 1 MCS was estimated to be approximately 36 seconds.

2 Model implementation

2.1 CC3D based cellular Potts model

The open source framework CompuCell3D (CC3D) was used to develop our multiscale computational model.[3] The package is based on highly modular design and provides the facility to incorporate the cell based cellular Potts model (or Graner-Glazier-Hogeweg(GGH)) Model)[4,5] and PDE based chemical reaction diffusion dynamics in a single framework. CC3D plugins, written in C++, were used to incorporate different energies including cell-motility, adhesion energies, area constraint, and perimeter constraint. Additionally, while all the plugins used in our model are part of the standard CC3D package, we wrote two steppables (python routines to add MCS level events) to incorporate aspects of ECM degradation and cell-polarity evolution and 2 steppables exclusively for visualization and data logging. Brief of these steppables is given below.

PolarityEvolutionSteppable: This steppable was developed to update the cell polarity after every MCS. As discussed in the material & method section and used in previous studies ,[1,6] the average displacement of cell during last 10 MCS ($D_{\text{avg},10}$) was quantified and then the new polarity of the cells was set equal to the direction of $D_{\text{avg},10}$. Initial polarities of all cells were set to be random. To integrate this polarity with cell-motility, the direction was multiplied by strength of motility (i.e., μ_0) and this vector was then supplied to the 'ExternalPotential' plugin (from python code only) which then implements the cell-motility ([3] and CC3D manual). This steppable also logs the centroid of all cells to CSV file for further analysis.

ECMDegradationSteppable: This steppable was developed to implement MMP mediated ECM degradation and was called after every MCS. This plugin checks the MMP concentration at all pixel positions and removes the fibre pixel (if any) from a pixel if the MMP concentration at that pixel location is ≥ 1.

IdFieldVisualizationSteppable: This steppable defines a cell level scaler field and assigns a random number to each cell. This

number can then be used to visualize an individual cell.

MMPSecretionSteppable: This steppable logs the temporal profile of total MMP in the lattice.

2.2 Custom tool for generating fibrillar matrix

To generate ECM lattice with varying fibre density (ξ) and orientation (ψ), a customized C++ program was written. Line drawing algorithm was used to generate ξ fibres, each of them $30-40\mu$m long and aligned at an angle ψ. The utility outputs the generated lattice in *.PIFF formate which then serves as input to the CC3D model using the 'PIFInitializer' steppable (part of standard CC3D package).

3 MMP activity of MCF-7 increases with increase in collagen density

MCF-7 cells (50×10^3 cells per cm^2) were grown in 500 μl of media on 3D collagen gels of varying densities (0.5, 1 and 2 mg/ml, respectively). After 36 hours of incubation, media were collected and centrifuged at 5000 rpm for 10 minutes to remove cells and gel particles (if any) and subjected to gelatin zymography.[7] In brief, equal volumes of media were mixed with 6X Laemmli non-reducing buffer, incubated for 15 minutes at room temperature. Samples were then electrophoresed on 8% SDS-PAGE gel containing 1 mg/ml gelatin. After electrophoresis, the gels were washed twice with renaturation buffer containing 2.5% triton X-100 for 30 minutes each, followed by rinsing 3 times for 30 minutes with reaction buffer containing 50 mM Tris-HCl buffer (pH 7.6) with 5 mM $CaCl_2$, 200 mM NaCl and incubated overnight at 37°C. The gels were stained with 0.5% Coomassie brilliant blue G-250 solution containing 10% acetic acid and 20% methanol for 30 min and destained with 7.5% acetic acid solution containing 10% methanol. Areas of gelatinase activity were detected as clear bands against the blue-stained gelatin background, which was quantified by densitometric analysis using ImageJ software. On analysis, we observed an increase in MMP (MMP-1, MMP-2 and MMP-9) activity with increase in collagen density (Figure S1).

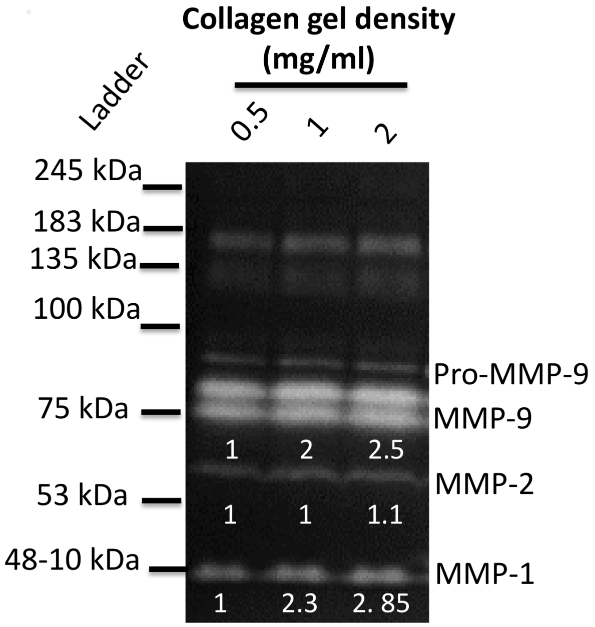

Figure S1. Increase in collagen density increases MMP secretion in MCF-7 breast cancer cells. Gelatin zymogram showing MMP-1, MMP-2, and MMP-9 activities from MCF-7 cells grown on three different collagen densities.

4 Supplementary Figures

4.1 Time scale of polarity evolution (τ) influences cell translocation

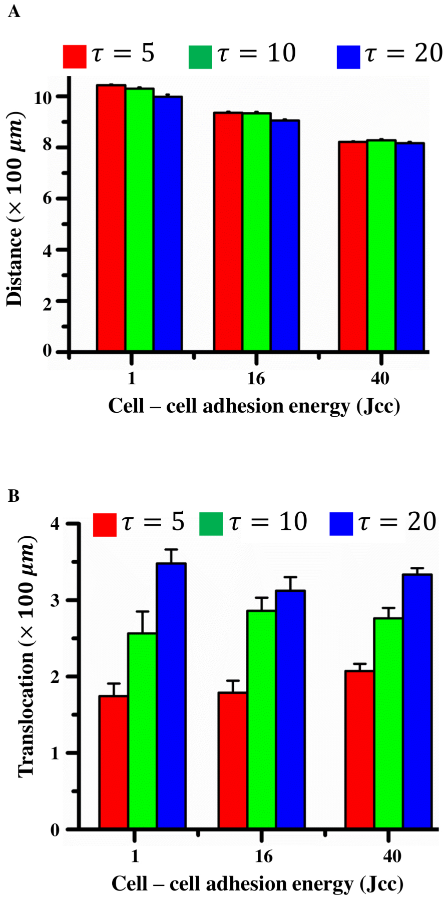

Figure S2. Increase in τ increases cell translocation. (A) Effect of τ on distance moved by cell for J_{cc} = 1, 16 and 40. (B) Effect of τ on total cell translocation for J_{cc} = 1, 16 and 40. Errorbar: ±SEM.

4.2 Analysis of displacement distribution for varying cell–cell adhesion

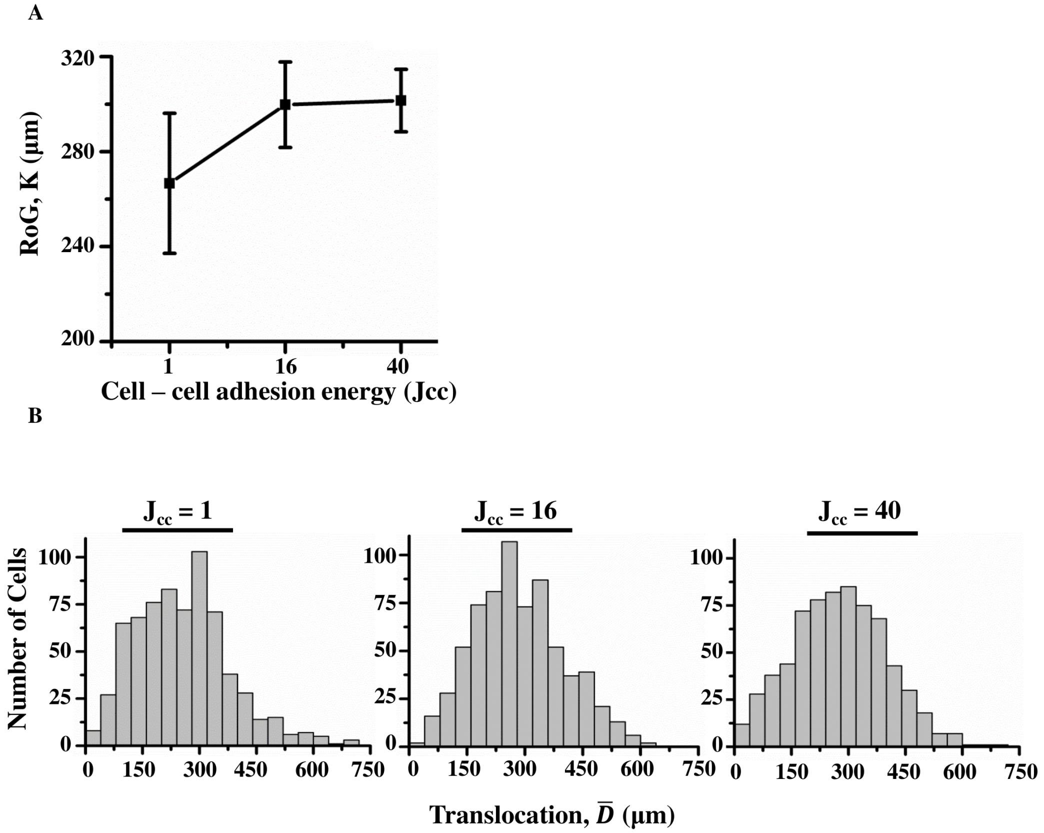

Figure S3. **Cell invasion at varying extents of cell–cell adhesion.** (A) Radius of gyration (RoG) for population of cells after 18 hours for varying cell–cell adhesion. (B) Distribution of translocation for all the 690 cells (i.e., 69 cells per simulation × 10 simulations for each J_{cc}) for different values of J_{cc}.

4.3 MMP secretion rate enhances cell scattering

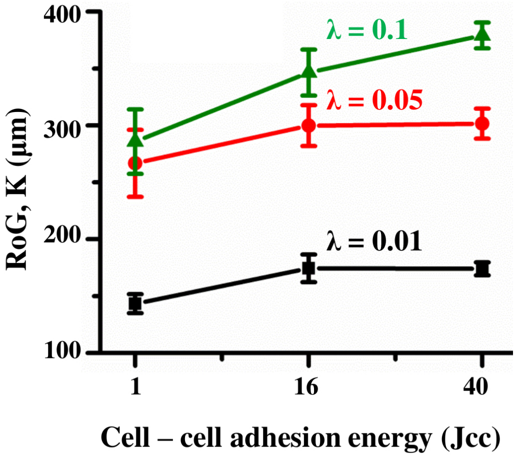

Figure S4. **Cell invasion with varying cell–cell adhesion (J_{cc}) and varying MMP secretion rate (λ).** Radius of gyration for population of cells after 18 hours for varying cell–cell adhesion.

4.4 Higher MMP secretion enhances collective cell invasion in dense matrices

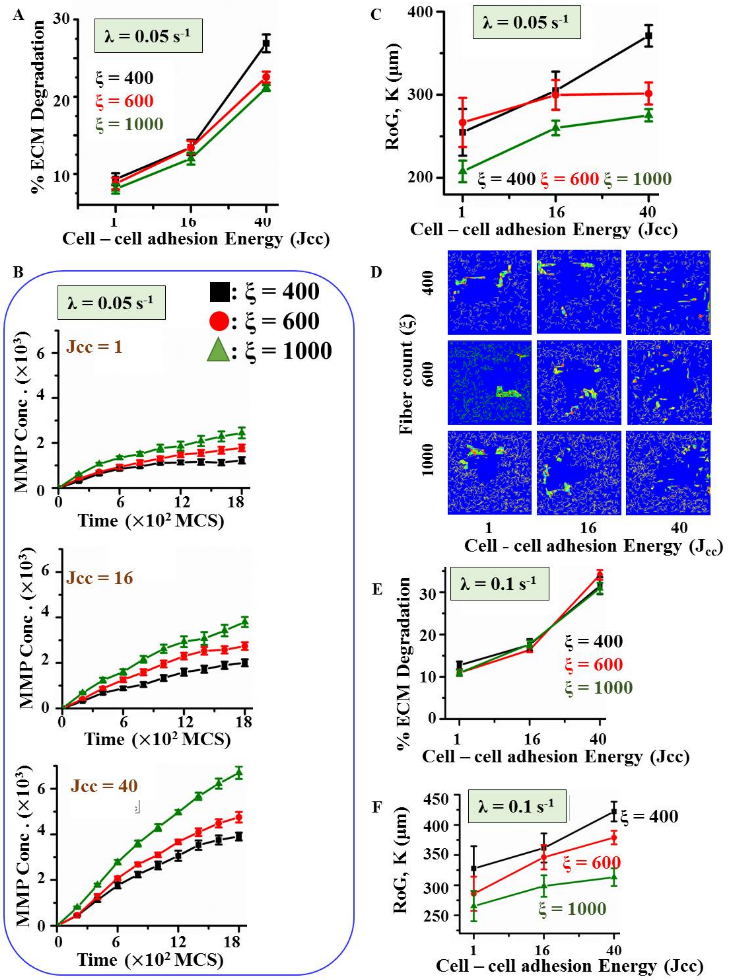

Figure S5. Higher MMP secretion enhances collective cell invasion in dense matrices (A) Percentage ECM degradation for moderate MMP secretion rate (i.e. $\lambda = 0.05$ s^{-1}) and varying cell–cell adhesion (J_{cc}) for different ECM densities (ξ). (B) Temporal profile of MMP count at moderate MMP secretion rate (i.e., $\lambda = 0.05$ s^{-1}) and $J_{cc} = 1, 16$ and 40. (C) Radius of gyration (RoG) for distribution of cells in population after 18 hours when simulations were performed with moderate MMP secretion rate (i.e., $\lambda = 0.05$ s^{-1}). (D) Representative images of invasion patterns when simulations were performed for different cell–cell adhesion and different ECM density at high MMP secretion rate (i.e., $\lambda = 0.1$ s^{-1}). (E) Percentage ECM degradation for higher MMP secretion rate (i.e. $\lambda = 0.1$ s^{-1}) and varying cell–cell adhesion (J_{cc}) for different ECM densities (ξ). (F) Radius of gyration (RoG) for distribution of cells in population after 18 hours for the case of high MMP secretion rate (i.e., $\lambda = 0.1$ s^{-1}).

4.5 Loss of cell–cell adhesion enhances cell invasion on aligned and dense matrices

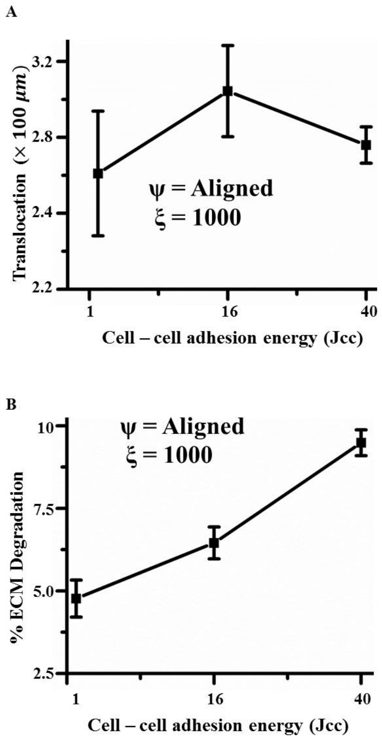

Figure S6. Loss of cell–cell adhesion enhances cell translocation and ECM degradation on aligned matrices. Cell invasion was simulated at the highest ECM density ($\xi = 1000$) and all aligned fibres to mimic a stiff matrix. Total cell translocation and Percentage ECM degradation was quantified after $\approx$ 18 hours. (A) Cell translocation for different extents of cell–cell adhesion ($i.e., J_{cc}$). (B) Percentage ECM degradation by cells with varying cell–cell adhesion capabilities. Errorbar: $\pm$SEM.

4.6 Aligned matrices sustain cell invasion independent of ECM proteolysis

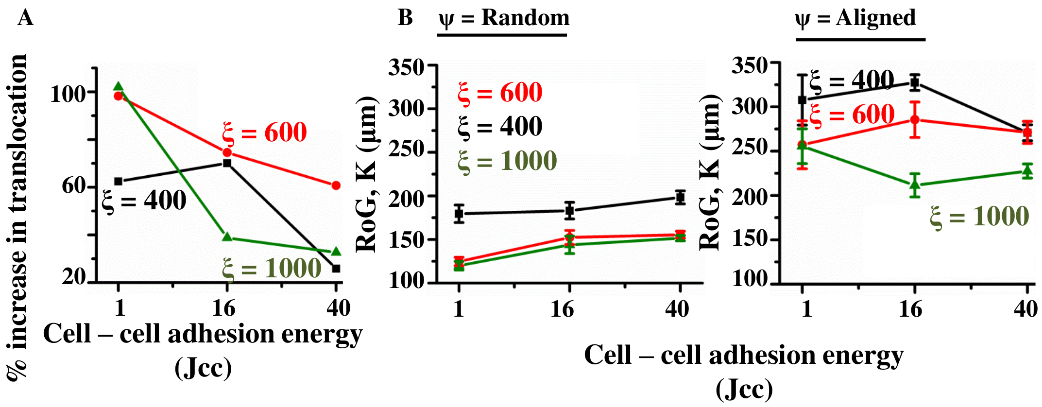

Figure S7. Aligned matrices can sustain cell invasion independent of ECM proteolysis. (A) Percentage increase in cell translocation when cells were placed in aligned matrices. Percentage increase was calculated with respect to the net translocation in random matrices. (B) Radius of gyration (RoG) for distribution of cells in population after 18 hours of simulation for random (Left) and aligned (Right) matrices. ECM proteolysis was turned off in these simulations.

4.7 Effect of ECM hindrance on cell translocation

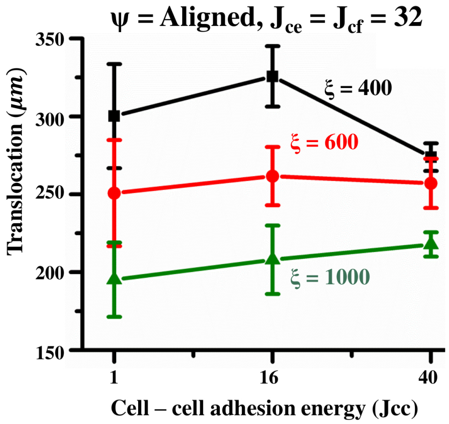

Figure S8. Effect of ECM hindrance on cell translocation. Simulations were performed with $J_{ce} = J_{cf}$, i.e., cells do not have any preference to attach to ECM fibres. Proteolysis was turned off in these simulations (i.e., $\lambda = 0$). Translocation of invading cells for varying cell–cell adhesion (J_{cc}) and ECM density (ξ).

5 Cell invasion in the presence of cell division

To study any potential influence of cell division on cell invasion, cells were allowed to divide. In all these simulations, cell division time was considered to be 24 hours (= 2400 MCS). To incorporate cell division into our model, 'MitosisSteppable' available with CompuCell3D was used.[3] Algorithmically, every cell was assigned an internal clock (*age*) to keep track of time (in hours) passed after the last division (Figure S9A). At $t = 0$, *age* of all cells were initialized to a random integer value $age \in [0, 24]$. After each 100 MCS ($\sim$1 hour), the value of *age* for each cell was incremented by one. Once the *age* of a cell reaches 24 hours, the cell was divided into two daughter cells and the *age* of daughter cells were reset to '0'. Random initialization of *age* was used to make sure that (i) some cells divided within 1800 MCS (otherwise they need atleast 2400 iterations), and (2) cell division happens asynchronously (i.e., all cells need not to divide at the same point of time). Due to this asynchronous cell proliferation, population size increased from 69 cells at $t = 0$ to $\sim$120 cells at the end of simulations ($t = 18$ hours).

Simulations were performed for varying cell–cell adhesions (i.e., J_{cc} = 1, 16 and 40), moderate ECM density (i.e., ξ = 600), moderate ECM proteolysis (i.e., $\lambda = 0.05\ s^{-1}$) and randomly aligned matrices (i.e., ψ = 'Random') (similar to Figure 2). For each value of J_{cc}, 10 simulations were performed. Radius of gyration (RoG), percentage ECM degradation and total MMP secretion were quantified after 1800 MCS. Since each cell division event introduces two new cells and removes the parent cell during the course of simulation, not all cells were present during the entire duration of simulation (1800 MCS). Hence, neither cell translocation ($\bar{D}$) after 18 hours (total simulation duration) nor the total distance traveled by a cell ($\bar{d}$) in 18 hours could be quantified. We, therefore, relied on radius of gyration (RoG) for quantifying cell scattering. Upon quantification of RoG, no drastic differences were observed when cell division was taken into account for $J_{cc} = 1, 16$; slight increase in RoG was observed for $J_{cc} = 40$ (Figure S9B). Mild increase in percentage ECM degradation and MMP secretion was observed in the presence of cell proliferation (Figure S9C and S9D). Since cell proliferation increases total cell population size, this mild increase in total MMP secretion and percentage ECM degradation was expected. Thus, these results suggest that cell proliferation does not appreciably change the attributes of cell invasion observed without taking cell division into account.

6 Cell invasion in the presence of cell supply

To study the effect of continuous supply of cells on cellular invasion, simulations were performed where cells were continuously added to the centre of the grid (where initial population was present). In this setup, the initial population represents a 2D slice of a 3D tumour where cells are continuously supplied from upper and lower layers of the 3D tumour. The rate of cell addition was kept proportional to the free space in the tumour centre (where initial population was present) (Figure S10A). Precisely, after every 10 MCS, $\frac{N_{\mathrm{FreePixels}}}{a_0}$ cells were added to the lattice centre, where $N_{\mathrm{FreePixels}}$ represents the number of free pixels and a_0 represents the number of target pixels (or target area in pixels) per cell (100 in our case). This continuous supply of cells caused a drastic increase in total number of cells in the population (> 200). Cell scattering (RoG) and percentage ECM

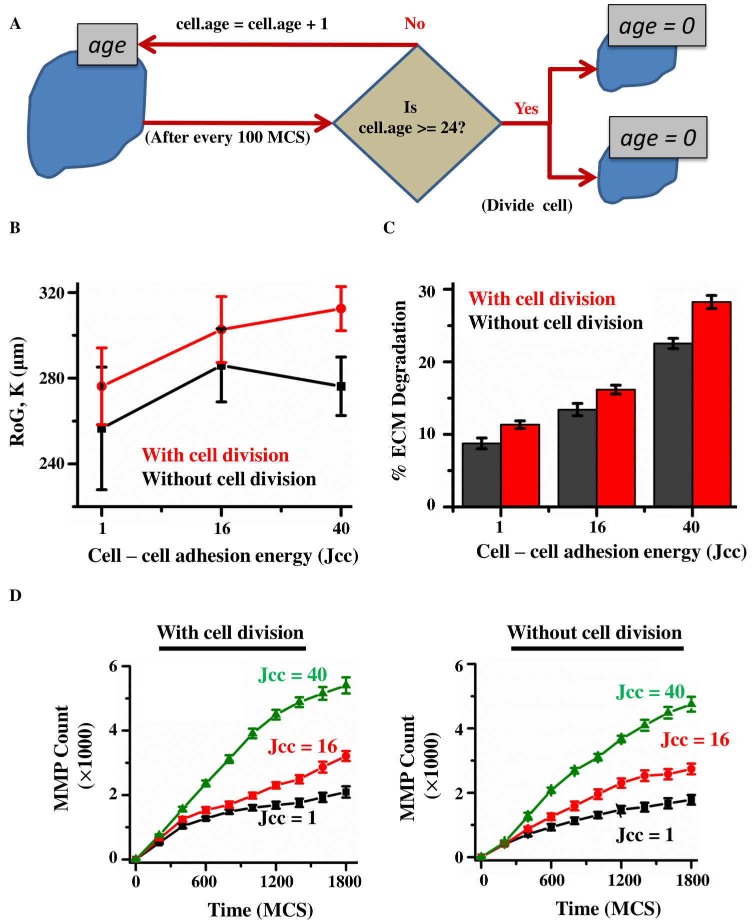

Figure S9. Influence of cell division on cell scattering, percentage ECM degradation and MMP secretion. (A) Schematic of cell addition. Each cell was associated with *age* clock. *age* of each cells was increased by 1 after every 100 MCS and the cell was divided if its *age* reaches 24. (B) Radius of gyration (RoG) for population of cells after 18 hours for varying cell–cell adhesion. (C) Percentage ECM degradation for different values of J_{cc}. (D) Temporal profile of total number of MMP molecules present in the lattice. Left: With cell division. Right: Without cell division.

degradation were quantified after 1000 MCS (instead of 1800 MCS), as beyond this time, due to their large number, cells started reaching the boundaries and moving along the boundaries. Temporal MMP profile was also traced for 1000 MCS. As expected, compared to the case of constant number of cells, higher levels of MMP secretion and percentage ECM degradation were observed for the case of continuous cell supply (Figure S10B and S10C). However, the behaviour of their their dependence on J_{cc} remained the same. While the dependency of RoG on J_{cc} differed between the two cases, it is difficult to make a direct comparison between the two cases. This is because for the case of continuous cell supply, cells are added at the centre thereby creating a bias in their spatial distribution, and hence influence the RoG measure differently (Figure S10D).

7 Supplementary Videos

Supplementary Video V1. **Rotational motion of cells confined in non-degradable circular geometry.** Video shows that when cells trapped in a circular confinement, they exhibit rotational motion. Simulation time is ≈ 18 hours. The circular geometry was designed using CellDraw utility available in CompuCell3D(CC3D) package.

Supplementary Video V2 - V4. **Cell invasion for varying cell–cell adhesion.** Video show the cell invasion with high cell–cell adhesion (i.e., J_{cc} = 1, Supp. Video V2), medium cell–cell adhesion (i.e., J_{cc} = 16, Supp. Video V3) and low cell–cell

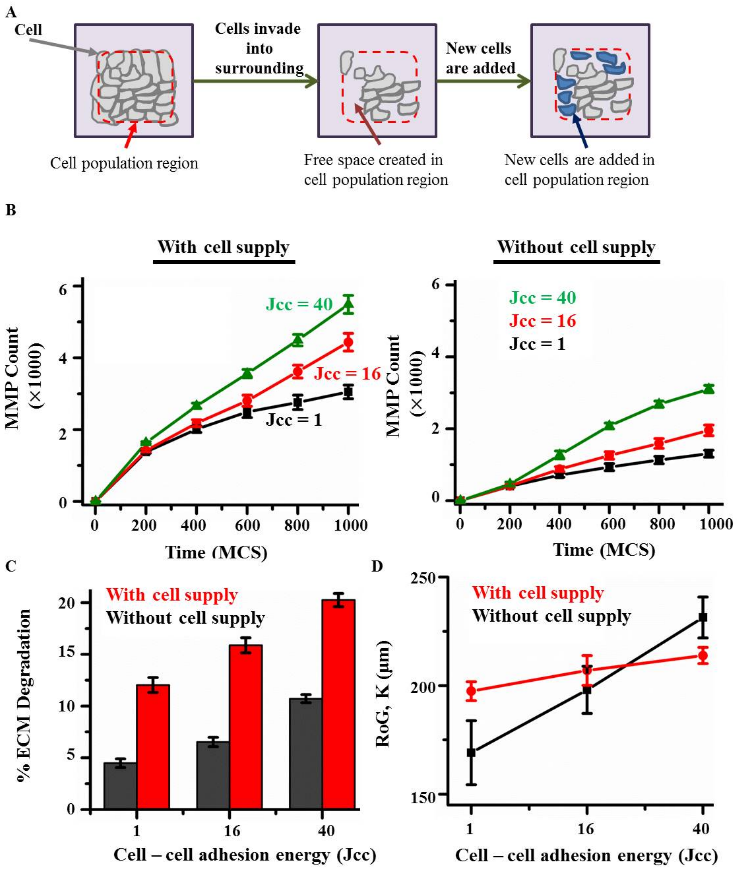

Figure S10. Influence of cell supply on MMP secretion, percentage ECM degradation and cell scattering. (A) Schematic of cell addition. As cells invade into the surroundings, new cells were added to the free space created. (B) Temporal profile of total number of MMP molecules present in the lattice for varying values of J_{cc}. Left: With cell supply. Right: Without cell supply. (C) percentage ECM degradation for different values of J_{cc}. (D) Radius of gyration (RoG) for population of cells after 10 hours.

adhesion (i.e., $J_{cc} = 40$, Supp. Vodeo V4). Duration of each movie was 18 hours.

Supplementary Video V5 - V8. **Proteolysis independent cell invasion in random matrices.** Videos show cell invasion when cells with different cell–cell adhesion abilities were allowed to invade through matrices composed of randomly aligned fibres at two different ECM densities. ECM degradation was turned off. **Video V5:** High cell–cell adhesion and low ECM density (i.e., $J_{cc} = 1$ and $\xi = 400$). **Video V6:** Low cell–cell adhesion and low ECM density (i.e., $J_{cc} = 40$ and $\xi = 400$). **Video V7:** High cell–cell adhesion and high ECM density (i.e., $J_{cc} = 1$ and $\xi = 1000$). **Video V8:** Low cell–cell adhesion and high ECM density (i.e., $J_{cc} = 40$ and $\xi = 1000$). Switching off of ECM proteolysis caused a significant drop in translocation for all combinations of cell–cell adhesion and ECM density.

Supplementary Videos V9 - V12. **Proteolysis independent cell invasion in aligned matrices.** Videos show cell invasion when cells with different cell–cell adhesion abilities were allowed to invade through aligned matrices at two different ECM densities. ECM degradation was turned off. **Video V9:** High cell–cell adhesion and low ECM density (i.e., $J_{cc} = 1$ and $\xi = 400$). **Video V10:** Low cell–cell adhesion and low ECM density (i.e., $J_{cc} = 40$ and $\xi = 400$). **Video V11:** High cell–cell adhesion and high ECM density (i.e., $J_{cc} = 1$ and $\xi = 1000$). **Video V12:** Low cell–cell adhesion and high ECM density (i.e., $J_{cc} = 40$ and $\xi = 1000$). A significant increase in cell translocation was observed in aligned matrices compared to random matrices (Supp. Videos V5 - V8). The exact increase in translocation was observed to be cell–cell adhesion dependent with maximum at highest cell–cell adhesion (i.e. $J_{cc} = 1$) (Figure 5C, Figure S7).

Supplementary Videos V13 and V14. **Migration of MCF-7 cells in sandwich collagen gels.** Videos show migration

of MCF-7 cells cultured in sandwich collagen gels. **Video V13:** Invasion when cell moves individually. Cell plating density: 5 $\times 10^3$ cells per cm^2. Collagen density: 0.5 mg/ml. **Video V14:** Invasion when cells move in group. Cell plating density: 25 $\times 10^3$ cell per cm^2. Collagen density: 0.5 mg/ml.

References

1. Doxzen, K. *et al.* Guidance of collective cell migration by substrate geometry. *Integr. Biol.* **5**, 1026–1035 (2013).
2. Vedula, S. R. K. *et al.* Emerging modes of collective cell migration induced by geometrical constraints. *Proc. Natl Acad. Sci. USA* **109**, 12974–9 (2012).
3. Swat, M. H. *et al.* Multi-scale modeling of tissues using CompuCell3D. *Meth. Cell Biol.* **110**, 325–366 (2012).
4. Graner, F. & Glazier, J. A. Simulation of biological cell sorting using a two-dimensional extended potts model. *Phys. Rev. Lett.* **69**, 2013 (1992).
5. Glazier, J. A. & Graner, F. Simulation of the differential adhesion driven rearrangement of biological cells. *Phys. Rev. E* **47**, 2128 (1993).
6. Kabla, A. J. Collective cell migration: leadership, invasion and segregation. *J. R. Soc. Interface* **9**, 3268–78 (2012).
7. Desai, S., Laskar, S. & Pandey, B. N. Autocrine IL-8 and VEGF mediate epithelial–mesenchymal transition and invasiveness via p38/JNK-ATF-2 signalling in A549 lung cancer cells. *Cellular signalling* **25**, 1780–1791 (2013).
